# Synthesis of β-Enaminonitrile-Linked 8-Methoxy-1*H*-Benzo[*f*]Chromene Moieties and Analysis of Their Antitumor Mechanisms

**DOI:** 10.3389/fchem.2021.759148

**Published:** 2021-11-22

**Authors:** Menna Elgaafary, Ahmed M. Fouda, Hany M. Mohamed, Abdelaaty Hamed, Heba K. A. El-Mawgoud, Lu Jin, Judith Ulrich, Thomas Simmet, Tatiana Syrovets, Ahmed M. El-Agrody

**Affiliations:** ^1^ Institute of Pharmacology of Natural Products and Clinical Pharmacology, Ulm University, Ulm, Germany; ^2^ Department of Pharmacognosy, College of Pharmacy, Cairo University, Cairo, Egypt; ^3^ Chemistry Department, Faculty of Science, King Khalid University, Abha, Saudi Arabia; ^4^ Chemistry Department, Faculty of Science, Jazan University, Jazan, Saudi Arabia; ^5^ Chemistry Department, Faculty of Science, Al-Azhar University, Cairo, Egypt; ^6^ Chemistry Department, Faculty of Women for Arts, Science, and Education, Ain Shams University, Cairo, Egypt

**Keywords:** 1H-benzo[f]chromenes, cell cycle, caspase 3/7, reactive oxygen species (ROS), mitochondrial membrane potential, structure–activity relationship, triple-negative breast cancer, chorioallantoic membrane (CAM) cancer xenografts 2

## Abstract

A series of aryl-substituted 3-amino-1-aryl-8-methoxy-1*H*-benzo[*f*]chromene-2-carbonitriles (4a–4q) were designed and synthesized via reaction of 6-methoxy-2-naphthol with a mixture of appropriate aromatic aldehydes and malononitrile under microwave conditions. The structures of the novel compounds 4b, 4c, 4f, 4g, 4i, 4l, 4m, and 4o–4q were established according to IR, ^1^H-NMR, ^13^C-NMR/^13^C-NMR-DEPT, and MS. The benzochromene derivative 4c with a single chlorine at the meta position of the phenyl ring and, to a lesser extent, other benzochromenes with monohalogenated phenyl ring (4a, 4c–4f) exhibited the highest cytotoxicity against six human cancer cell lines MDA-MB-231, A549, HeLa, MIA PaCa-2, 5,637, and Hep G2. The mechanisms of the cytotoxic activities of benzochromenes with monohalogenated phenyl ring (4a, 4c–4f) were further analyzed using triple-negative breast cancer cell line MDA-MB-231. Cell cycle analysis showed accumulation of the treated cells in S phase for 4a, 4d–4f, and S-G_2_/M phases for 4c. *In vivo*, 4a and 4c–4f inhibited growth, proliferation, and triggered apoptosis in preestablished breast cancer xenografts grown on the chick chorioallantoic membranes while exhibiting low systemic toxicity. Compounds 4a and 4c–4f increased levels of mitochondrial superoxide and decreased mitochondrial membrane potential resulting in initiation of apoptosis as demonstrated by caspase 3/7 activation. In addition, 4c induced general oxidative stress in cancer cells. The SAR study confirmed that halogens of moderate size at meta or para positions of the pendant phenyl ring enhance the cytotoxic activity of 3-amino-1-aryl-8-methoxy-1*H*-benzo[*f*]chromene-2-carbonitriles, and these compounds could serve as leads for the development of novel anticancer therapies.

## Introduction

Cancer is still one of the leading cause of death and among the major public health concerns (Sung et al., 2021). In 2020, an estimated 19.3 million new cancer cases and almost 10.0 million cancer deaths occurred worldwide, and the global cancer burden is expected to rise by 47% in 2040 (Sung et al., 2021). In males, lung cancer is the most frequently diagnosed cancer type and the leading cause of cancer-related death, followed by prostate for incidence and liver for mortality. In females, breast cancer is the most common type of cancer and the leading cause of cancer-related mortality (Torre et al., 2015; Sung et al., 2021). Triple-negative breast cancer (TNBC) is a breast cancer subtype that is more likely to be diagnosed in younger premenopausal women. TNBC is of special concern due to its aggressive character and to the lack of effective targeted therapies (Carey et al., 2010; Lee et al., 2011; Park et al., 2018). Therefore, there is a general need for the development of new approaches to efficiently control these diseases.

Chromene and benzochromene scaffolds are well known as important components of biologically active synthetic or natural compounds. Indeed, these compounds display a wide variety of biological activities (Costa et al., 2016). Moreover, the lipophilic properties of the benzopyran structures enable efficient transport of these compounds across biological membranes (Nicolaou et al., 2000). Some of these molecules exhibit potential therapeutic activities, which can be exploited in the treatment of cancer, and infectious and inflammatory diseases. In addition, the above properties of chromene derivatives might be useful for the treatment of different neurological disorders; among them, depression, schizophrenia, and Alzheimer’s disease have been considered (Patil et al., 2013; Costa et al., 2016).

**FIGURE 1:**
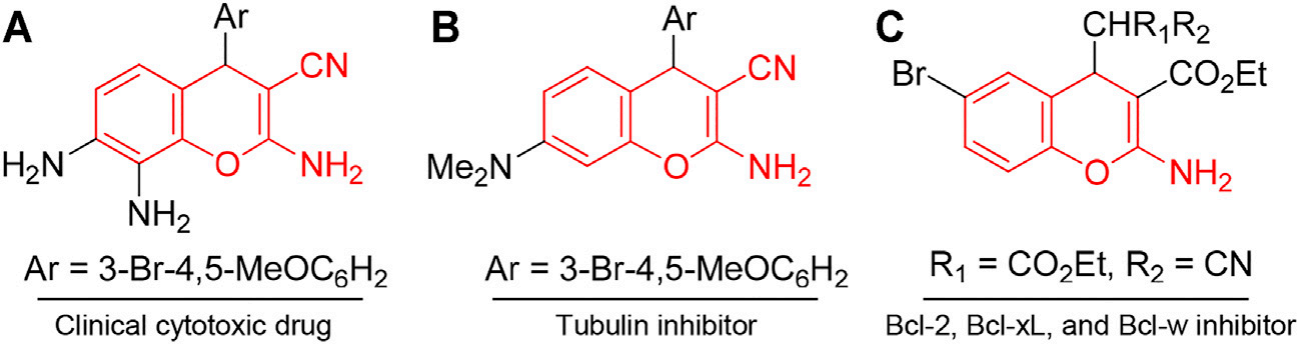
Compounds with chromene scaffold (highlighted in red) exhibiting cytotoxic effects.

The chromene unit constitutes the basic structural framework of many natural products such as coumarins and polyphenols, although those natural products are difficult to obtain in commercial quantities. Thus, different chromene systems have been synthesized with favorable and desirable scaffolds for the development of potent cytotoxic agents. For example, Crolibulin™ [2,7,8-triamino-4-(3-bromo-4,5-dimethoxyphenyl)-4*H*-chromene-3-carbonitrile, A] has progressed to the NCI phase I/II clinical studies for the treatment of patients with advanced solid tumors (Gramza et al., 2013; Patil et al., 2013). Crolibulin™ alone, or in combination with cisplatin, demonstrated favorable safety and tolerability profiles in phase I clinical study. The results were encouraging and fully supported the conduction of further clinical trials (Gramza et al., 2013; Patil et al., 2013). MX-58151 (2-Amino-4-(3-bromo-4,5-dimethoxyphenyl)-7-(dimethylamino)-4*H*-chromene-3-carbonitrile, B) and related compounds act as tubulin destabilizers (Kasibhatla et al., 2004), and HA14-1 [ethyl 2-amino-6-bromo-4-(1-cyano-2-ethoxy-2-oxoethyl)-4*H*-chromene-3-carboxylate, C] and its analogs act as inhibitors of the antiapoptotic Bcl-2, Bcl-xL, and Bcl-w proteins and inducers of apoptosis (Figure 1) (Wang et al., 2000; Doshi et al., 2006).

**FIGURE 2:**
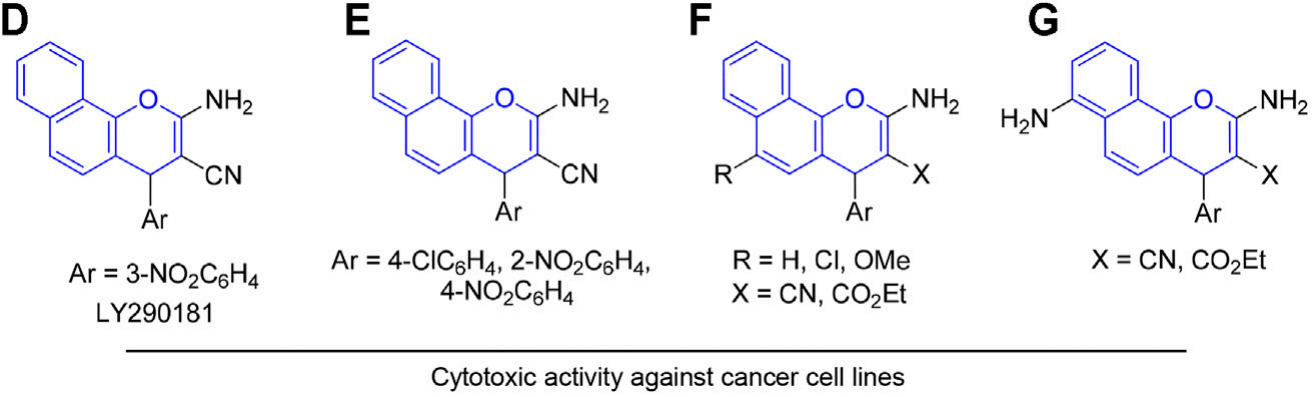
Compounds with 4H-benzo[h]chromene scaffold (highlighted in blue) exhibiting cytotoxic effects.

In addition, derivatives of 4*H*-benzo[*h*]chromene represent promising scaffolds for the development of potential anticancer agents. For instance, 2-amino-4-(3-nitrophenyl)-4*H*-benzo[*h*]chromene-3-carbonitrile (LY290181) (D) inhibited the proliferation of mammalian cells at prometaphase by an apparent action on microtubules (Panda et al., 1997; Wood et al., 1997), and aryl-substituted derivatives of 2-amino-4-aryl-4*H*-benzo[*h*]chromene-3-carbonitrile (E) exhibited cytotoxic and proapoptotic effects on a variety of human cancer cell lines (Kheirollahi et al., 2014). 2-Amino-4-aryl-4*H*-benzo[*h*]chromene-3-carbonitriles, ethyl 2-amino-4-aryl-4*H*-benzo[*h*]chromene-3-carboxylates, and their 6-chloro/-methoxy derivatives (F) (El-Agrody et al., 2013; El-Agrody et al., 2014; Halawa et al., 2016; El-Agrody et al., 2017a; El-Agrody et al., 2017b), in addition to 4-aryl-2,7-diamino-4*H*-benzo[*h*]chromene-3-carbonitrile (G), ethyl 4-aryl-2,7-diamino-4*H*-benzo[*h*]chromene-3-carboxylate (El-Agrody et al., 2016), and their related compounds have been reported to exert cytotoxic activities against human breast adenocarcinoma (MCF7), human colon carcinoma (HCT-116), and human hepatocellular carcinoma (Hep G2) cells (Figure 2).

**FIGURE 3:**
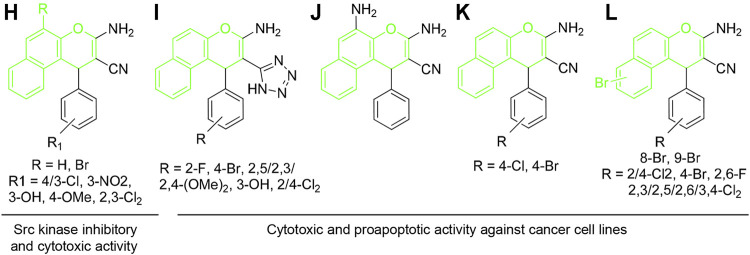
Compounds with 4H-benzo[f]chromene scaffold (highlighted in green) exhibiting cytotoxic effects.

Similarly, 1*H*-benzo[*f*]chromene derivatives are regarded as promising lead candidates for anticancer drug development. For example, some derivatives of 3-amino-1-aryl-1*H*-benzo[*f*]chromene-2-carbonitrile including 3-amino-8-bromo-1-(4-chlorophenyl)-1*H*-benzo[*f*]chromene-2-carbonitrile (H) exhibited c-Src kinase inhibitory and proapoptotic activities (Ahmed et al., 2018). A series of 1-substituted aryl-2-(1H-tetrazol-5-yl)-1*H*-benzo[*f*]chromene-3-amines (I), some derivatives of 3,5-diamino-1-phenyl-1*H*-benzo[*f*]chromene-2-carbonitrile including 3,5-diamino-1-(p-tolyl)-1*H*-benzo[*f*]chromene-2-carbonitrile (J), and 3-amino-1-(4-chloro/-bromophenyl)-1*H*-benzo[*f*]chromene-2-carbonitriles (K) have been reported to exhibit cytotoxic and apoptotic effects against a variety of human cancer cell lines (Afifi et al., 2017; Gorle et al., 2017; Kheirollahi et al., 2014). Aryl-substituted derivatives of 3-amino-8/9-bromo-1-aryl-1*H*-benzo[*f*]-chromene-2-carbonitrile (L) have been shown to induce cell cycle arrest and apoptosis in human cancer cells via dual inhibition of topoisomerases and tubulin (Figure 3) (Fouda et al., 2019; Fouda et al., 2020).

Considering low systemic toxicity and diverse biological activities of benzochromene derivatives, we have used the 1*H*-benzo[*f*]chromene scaffold with methoxy substituent at the 8-position and different halogen substituents on the pendant phenyl group at the 1-position of 1*H*-benzo[*f*]chromene moiety to create various compounds. The cytotoxic activities of the synthesized compounds were examined using cancer cell lines that represent different advanced human malignancies. In addition, intracellular effects and intracellular targets of benzochromene compounds with mono-meta/para-halogenated-substituted phenyl ring (4a, 4c–4f), which exhibited the most pronounced cytotoxicity against the human cancer cell lines tested, were analyzed *in vitro* and *in vivo*.

## Materials and methods

### General Procedures

All chemicals were purchased from Sigma-Aldrich (St. Louis, MO, USA). All melting points were determined on a Stuart™ digital melting point apparatus (Stone, UK) and are uncorrected. The optical rotation was measured using Carl Zeiss polarimeter (Carl Zeiss, Oberkochen, Germany). The IR spectra were recorded on a Jasco FT/IR 460 plus spectrophotometer using KBr discs. The ^1^H-NMR (500 MHz) and ^13^C-NMR (125 MHz) spectra were recorded on BRUKER AV 500 MHz spectrometer (Billerica, MA) in DMSO-d_6_ as a solvent, using tetramethylsilane (TMS) as an internal standard, and chemical shifts were expressed as *δ* (ppm). The microwave apparatus used is Milestone Sr1, Microsynth (Balgach, Switzerland). The mass spectra were measured using Shimadzu GC/MS-QP5050A spectrometer (Kyoto, Japan). Elemental analysis was carried out at the Regional Centre for Mycology and Biotechnology (RCMP, Al-Azhar University, Cairo, Egypt), and the results were within ± 0.25%. Reaction courses, product mixtures, and purity of compounds were monitored by thin layer chromatography (TLC) performed on Merck precoated silica gel 60 F254 aluminum sheets.

### General Procedure for the Synthesis of 1*H*-Benzo[*f*]Chromene Derivatives (4a–4q)

A reaction mixture of 6-methoxy-2-naphthol (1) (0.01 mol), different aromatic aldehydes (2a-2q) (0.01 mol), malononitrile (3) (0.01 mol), and piperidine (0.5 ml) in ethanol (25 ml) was heated under microwave irradiation conditions at 140°C for 2 min. After completion of the reaction, the reaction mixture was cooled to room temperature, and the precipitated solid was filtered off, washed with methanol, and recrystallized from suitable solvent. The physical and spectral data of compounds 4a–4q are as follows:

3-Amino-1-(4-fluorophenyl)-8-methoxy-1*H*-benzo[*f*]chromene-2-carbonitrile (4a). Compound 4a was prepared according to the literature procedure (Ahmed et al., 2018).

3-Amino-1-(2-chlorophenyl)-8-methoxy-1*H*-benzo[*f*]chromene-2-carbonitrile (4b). Colorless needles from ethanol; yield 82%; m.p. 265–266°C; IR (KBr) *υ* (cm^−1^): 3,407, 3,330, 3,210 (NH_2_), 2,200 (CN); ^1^H-NMR *δ*: 7.87–6.99 (m, 9H, aromatic), 7.00 (bs, 2H, NH_2_), 5.67 (s, 1H, H-1), 3.82 (s, 3H, OCH_3_); ^13^C-NMR *δ*: 159.96 (C-3), 156.49 (C-8), 145.64 (C-4a), 130.97 (C-6a), 129.50 (C-10a), 128.15 (C-6), 125.01 (C-10), 119.87 (C-10b), 119.35 (C-9), 117.09 (C-7), 114.92 (CN), 107.57 (C-5), 56.11 (C-2), 55.21 (CH_3_), 35.17 (C-1), 142.66, 132.14, 130.00, 128.58, 128.44, 124.09 (aromatic); MS m/z (%): 364 (M^+^ + 2, 4.65), 362 (M^+^, 13.54) with a base peak at 251 (100); Anal. Calcd for C_21_H_15_ClN_2_O_2_ (362.81): C, 69.52; H, 4.17; N, 7.72. Found: C, 69.47; H, 4.13; N, 7.68%.

3-Amino-1-(3-chlorophenyl)-8-methoxy-1*H*-benzo[*f*]chromene-2-carbonitrile (4c). Colorless needles from ethanol; yield 86%; m.p. 215–216°C; IR (KBr) *υ* (cm^−1^): 3,468, 3,324, 3,198 (NH_2_), 2,197 (CN); ^1^H-NMR *δ*: 7.87–7.11 (m, 9H, aromatic), 7.03 (bs, 2H, NH_2_), 5.36 (s, 1H, H-1), 3.83 (s, 3H, OCH_3_); ^13^C-NMR *δ*: 159.94 (C-3), 156.52 (C-8), 148.19 (C-4a), 132.21 (C-6a), 128.48 (C-10a), 126.63 (C-6), 124.99 (C-10), 120.31 (C-10b), 119.21 (C-9), 117.11 (C-7), 115.11 (CN), 107.35 (C-5), 57.18 (C-2), 55.20 (CH_3_), 37.60 (C-1), 145.36, 133.18, 130.66, 126.61, 125.65 (aromatic); MS m/z (%): 364 (M^+^+ 2, 1.24), 362 (M^+^, 3.58) with a base peak at 208 (100); Anal. Calcd for C_21_H_15_ClN_2_O_2_ (362.81): C, 69.52; H, 4.17; N, 7.72. Found: C, 69.57; H, 4.23; N, 7.77%.

3-Amino-1-(4-chlorophenyl)-8-methoxy-1*H*-benzo[*f*]chromene-2-carbonitrile (4d). Compound 4d was prepared according to the literature procedure (Ahmed et al., 2018).

3-Amino-1-(4-bromophenyl)-8-methoxy-1*H*-benzo[*f*]chromene-2-carbonitrile (4e). Compound 4e was prepared according to the literature procedure (Ahmed et al., 2018).

3-Amino-1-(4-iodophenyl)-8-methoxy-1*H*-benzo[*f*]chromene-2-carbonitrile (4f). Pale yellow needles from ethanol; yield 89%; m.p. 227–228°C; IR (KBr) *υ* (cm^−1^): 3,455, 3,322, 3,190 (NH_2_), 2,201 (CN); ^1^H-NMR *δ*: 7.85–6.99 (m, 9H, aromatic), 6.98 (bs, 2H, NH_2_), 5.27 (s, 1H, H-1), 3.82 (s, 3H, OCH_3_); ^13^C-NMR *δ*: 159.80 (C-3), 156.48 (C-8), 145.31 (C-4a), 132.20 (C-6a), 128.37 (C-10a), 125.01 (C-6), 120.37 (C-10), 119.11 (C-10b), 117.08 (C-7,9), 115.22 (CN), 107.35 (C-5), 57.29 (C-2), 55.21 (CH_3_), 37.69 (C-1), 145.54, 137.43, 129.32, 92.40 (aromatic); MS m/z (%): 454 (M^+^, 100); Anal. Calcd for C_21_H_15_IN_2_O_2_ (454.26): C, 55.52; H, 3.33; N, 6.17. Found: C, 55.65; H, 3.46; N, 6.34%.

3-Amino-1-(2,4-difluorophenyl)-8-methoxy-1*H*-benzo[*f*]chromene-2-carbonitrile (4g). Colorless crystals from ethanol; yield 81%; m.p. 301–302°C; IR (KBr) *υ* (cm^−1^): 3,476, 3,335, 3,291 (NH_2_), 2,201 (CN); ^1^H-NMR *δ*: 7.83–7.05 (m, 8H, aromatic), 7.09 (bs, 2H, NH_2_), 5.67 (s, 1H, H-1), 3.82 (s, 3H, OCH_3_); ^13^C-NMR *δ*: 160.71 (C-3), 149.61 (C-8), 145.61 (C-4a), 131.44 (C-6a), 129.11 (C-10a), 128.39 (C-6), 123.52 (C-10), 120.18 (C-9), 120.04 (C-7), 119.92 (C-10b), 116.94 (CN), 107.57 (C-5), 55.19 (C-2), 53.58 (CH_3_), 28.39 (C-1), 161.40, 156.14, 129.39, 125.59, 116.64, 112.82 (aromatic); MS m/z (%): 364 (M^+^, 61.46) with a base peak at 251 (100); Anal. Calcd for C_21_H_14_F_2_N_2_O_2_ (364.34): C, 69.23; H, 3.87; N, 7.69. Found: C, 69.29; H, 3.92; N, 7.73%.

3-Amino-1-(2,6-difluorophenyl)-8-methoxy-1*H*-benzo[*f*]chromene-2-carbonitrile (4h). Compound 4h was prepared according to the literature procedure (Halawa et al., 2017).

3-Amino-1-(2,3-dichlorophenyl)-8-methoxy-1*H*-benzo[*f*]chromene-2-carbonitrile (4i). Colorless needles from ethanol; yield 87%; m.p. 255–256°C; IR (KBr) *υ* (cm^−1^): 3,423, 3,335, 3,206 (NH_2_), 2,196 (CN); ^1^H-NMR *δ*: 7.88–6.96 (m, 8H, aromatic), 7.07 (bs, 2H, NH_2_), 5.74 (s, 1H, H-1), 3.83 (s, 3H, OCH_3_); ^13^C-NMR *δ*: 160.08 (C-3), 156.53 (C-8), 145.23 (C-4a), 131.98 (C-6a), 129.16 (C-10a), 128.79 (C-6), 124.92 (C-10), 119.77 (C-10b), 119.50 (C-9), 117.09 (C-7), 114.44 (CN), 107.65 (C-5), 55.59 (C-2), 55.21 (CH_3_), 36.22 (C-1), 145.63, 132.16, 129.01, 128.93, 128.64, 123.95 (aromatic); MS m/z (%): 400 (M^+^+ 4, 10.10), 398 (M^+^+ 2, 3.37), 396 (M^+^, 12.08) with a base peak at 110 (100); Anal. Calcd for C_21_H_14_Cl_2_N_2_O_2_ (397.25): C, 63.49; H, 3.55; N, 7.05. Found: C, 63.54; H, 3.60; N, 7.10%.

3-Amino-1-(2,4-dichlorophenyl)-8-methoxy-1*H*-benzo[*f*]chromene-2-carbonitrile (4j). Compound 4j was prepared according to the literature procedure (Okasha et al., 2017).

3-Amino-1-(2,5-dichlorophenyl)-8-methoxy-1*H*-benzo[*f*]chromene-2-carbonitrile (4k). Compound 4k was prepared according to the literature procedure (El Gaafary et al., 2021).

3-Amino-1-(2,6-dichlorophenyl)-8-methoxy-1*H*-benzo[*f*]chromene-2-carbonitrile (4l). Colorless crystals from ethanol; yield 85%; m.p. 314–315°C; IR (KBr) *υ* (cm^−1^): 3,466, 3,318, 3,198 (NH_2_), 2,193 (CN); ^1^H-NMR *δ*: 7.84–7.09 (m, 8H, aromatic), 7.05 (bs, 2H, NH_2_), 6.09 (s, 1H, H-1), 3.82 (s, 3H, OCH_3_); ^13^C-NMR *δ*: 160.41 (C-3), 156.22 (C-8), 146.39 (C-4a), 134.33 (C-6a), 132.01 (C-10a), 128.86 (C-6), 125.22 (C-10), 123.83 (C-10b), 119.56 (C-9), 119.18 (C-7), 116.78 (CN), 107.69 (C-5), 55.18 (C-2), 52.59 (CH_3_), 35.19 (C-1), 137.45, 135.04, 130.96, 129.57 (aromatic); MS m/z (%): 400 (M^+^+ 4, 6.19), 398 (M^+^+ 2, 2.09), 396 (M^+^, 7.88) with a base peak at 251 (100); Anal. Calcd for C_21_H_14_Cl_2_N_2_O_2_ (397.25): C, 63.49; H, 3.55; N, 7.05. Found: C, 63.46; H, 3.52; N, 7.02%.

3-Amino-1-(3,4-dichlorophenyl)-8-methoxy-1*H*-benzo[*f*]chromene-2-carbonitrile (4m). Colorless crystals from ethanol/benzene; yield 85%; m.p. 240–241°C; IR (KBr) *υ* (cm^−1^): 3,412, 3,328, 3,200 (NH_2_), 2,198 (CN); ^1^H-NMR *δ*: 7.88–6.96 (m, 8H, aromatic), 7.08 (bs, 2H, NH_2_), 5.76 (s, 1H, H-1), 3.84 (s, 3H, OCH_3_); ^13^C-NMR *δ*: 160.08 (C-3), 156.53 (C-8), 145.63 (C-4a), 129.01 (C-6a), 128.79 (C-10a), 128.64 (C-6), 123.95 (C-10), 119.77 (C-10b), 119.50 (C-9), 117.09 (C-5), 114.44 (CN), 107.65 (C-7), 55.59 (C-2), 55.21 (CH_3_), 36.23 (C-1), 145.23, 132.16, 131.98, 129.15, 128.93, 128.79, 124.92 (aromatic); MS m/z (%): 400 (M^+^+ 4, 1.49), 398 (M^+^+ 2, 0.47), 396 (M^+^, 1.79) with a base peak at 208 (100); Anal. Calcd for C_21_H_14_Cl_2_N_2_O_2_ (397.25): C, 63.49; H, 3.55; N, 7.05. Found: C, 63.46; H, 3.52; N, 7.02%.

3-Amino-1-(3,5-dibromo-2-methoxyphenyl)-8-methoxy-1*H*-benzo[*f*]chromene-2-carbonitrile (4n). Compound 4n was prepared according to the literature procedure (Amr et al., 2017).

3-Amino-1-(2,3,4-trimethoxyphenyl)-8-methoxy-1*H*-benzo[*f*]chromene-2-carbonitrile (4o). Colorless crystals from ethanol; yield 84%; m.p. 239–240°C; IR (KBr) *υ* (cm^−1^): 3,424, 3,381, 3,322 (NH_2_), 2,192 (CN); ^1^H-NMR *δ*: 7.77–6.55 (m, 7H, aromatic), 6.94 (bs, 2H, NH_2_), 5.31 (s, 1H, H-1), 3.80 (s, 3H, OCH_3_), 3.78 (s, 6H, 2OCH_3_), 3.71 (s, 3H, OCH_3_); ^13^C-NMR *δ*: 159.98 (C-3), 156.79 (C-8), 145.65 (C-4a), 131.83 (C-10a), 128.25 (C-6), 125.74 (C-6a), 125.03 (C-10), 121.54 (C-10b), 119.39 (C-7), 117.59 (C-9), 116.88 (CN), 107.62 (C-5), 60.91 (CH_3_), 60.54 (C-2), 57.53 (C-2), 55.98 (CH_3_), 55.41 (CH_3_), 33.01 (C-1), 152.86, 140.92, 136.11, 112.64, 104.62 (aromatic); MS m/z (%): 418 (M^+^, 77.17) with a base peak at 387 (100); Anal. Calcd for C_24_H_22_N_2_O_5_ (418.44): C, 68.89; H, 5.30; N, 6.69. Found: C, 68.81; H, 5.29; N, 6.61%.

3-Amino-1-(3,4,5-trimethoxyphenyl)-8-methoxy-1*H*-benzo[*f*]chromene-2-carbonitrile (4p). Colorless crystals from ethanol; yield 84%; m.p. 259–260°C; IR (KBr) *υ* (cm^−1^): 3,469, 3,350, 3,225 (NH_2_), 2,197 (CN); ^1^H-NMR *δ*: 7.78–6.60 (m, 7H, aromatic), 6.92 (bs, 2H, NH_2_), 5.35 (s, 1H, H-1), 3.81 (s, 3H, OCH_3_), 3.79 (s, 3H, OCH_3_), 3.72 (s, 3H, OCH_3_), 3.66 (s, 3H, OCH_3_); ^13^C-NMR *δ*: 160.60 (C-3), 156.80 (C-8), 145.70 (C-4a), 131.85 (C-10a), 128.23 (C-6), 125.75 (C-6a), 125.00 (C-10), 121.53 (C-10b), 119.46 (C-7), 117.55 (C-9), 116.86 (CN), 107.60 (C-5), 61.75 (CH_3_), 60.62 (CH_3_), 57.62 (C-2), 56.05 (CH_3_), 55.57 (CH_3_), 33.04 (C-1), 152.49, 150.24, 141.71, 132.45, 123.80, 108.77 (aromatic); ^13^C-NMR-DEPT spectrum at 135°: CH, CH_3_ [positive (up)] and CH_2_ [negative (down)], revealed the following signals at *δ*: 128.23 (C-6 ↑), 125.00 (C-10 ↑), 123.80 (aromatic ↑), 119.46 (C-7 ↑), 117.55 (C-9 ↑), 108.77 (aromatic ↑), 107.60 (C-5 ↑), 61.75 (CH_3_ ↑), 60.62 (CH_3_ ↑), 56.05 (CH_3_ ↑), 55.57 (CH_3_ ↑), 33.04 (C-1 ↑); MS m/z (%): 418 (M^+^, 13.93) with a base peak at 40 (100); Anal. Calcd for C_24_H_22_N_2_O_5_ (418.44): C, 68.89; H, 5.30; N, 6.69. Found: C, 68.95; H, 5.37; N, 6.76%.

3-Amino-1-(2,3,5-trichlorophenyl)-8-methoxy-1*H*-benzo[*f*]chromene-2-carbonitrile (4q). Colorless crystals from ethanol; yield 84%; m.p. 259–260°C; IR (KBr) *υ* (cm^−1^): 3,441, 3,333, 3,210 (NH_2_), 2,183 (CN); MS m/z (%): 436 (M^+^+ 6, 3.91), 434 (M^+^+ 4, 33.80), 432 (M^+^+ 2, 96.27), 430 (M^+^, 100); Anal. Calcd for C_21_H_13_Cl_3_N_2_O_2_ (431.70): C, 58.43; H, 3.04; N, 6.49. Found: C, 58.49; H, 3.10; N, 6.54%.

### Cell Lines

All cell lines were purchased from the American Type Culture Collection (ATCC, Rockville, MD, USA), except for the human urinary bladder carcinoma cell line 5,637, which was obtained from the German Collection of Microorganisms and Cell Cultures (DSMZ, Braunschweig, Germany) and cultured as recommended. Cells were used in all experiments without synchronization.

### Cell Viability Analysis

The XTT cell viability assay (Roche Diagnostics, Filderstadt, Germany) measures cellular metabolic activity. Cells were treated with various concentrations of the respective compounds for 24 h and analyzed as recommended by manufacturer using a TECAN Infinite^®^ 200 PRO microplate reader (Tecan, Männedorf, Switzerland) (El Gaafary et al., 2017; El Gaafary et al., 2019). Etoposide (Sigma-Aldrich), the broad-spectrum anticancer drug, served as positive control. The results are expressed as IC_50_ (concentration leading to half-maximal inhibition) and GR_50_ (concentration leading to half-maximal growth inhibition), alternative small-molecule drug-response metrics (Hafner et al., 2016).

### Cell Cycle Analysis

Following a 24-h treatment of MDA-MB-231 cells with either the most active benzochromene derivatives (4a, 4c–4f), etoposide, or camptothecin (Calbiochem, Merck, Darmstadt, Germany), all at 10 μM, cells were permeabilized, stained with propidium iodide, and analyzed by using flow cytometry and FACSVerse cytometer (BD Biosciences, Franklin Lakes, NJ, USA) (Nicoletti et al., 1991). Further analysis was performed with FlowJo software (TreeStar Inc., Ashland, OR).

### Analysis of Mitochondrial Integrity and Mitochondrial Superoxide Generation

MDA-MB-231 cells were treated with 4a, 4c–4f at a concentration of 10 µM for 24 h. The treated cells were loaded with 10 μg/ml of the voltage-sensitive lipophilic cationic probe JC-1 (Molecular Probes, San Diego, CA, USA) in serum-free medium for 20 min at 37°C, excited by the 488-nm laser wavelength, and analyzed at 527 and 590 nm by using flow cytometry (El Gaafary et al., 2015; El Gaafary et al., 2017). Cells treated with the mitochondrial uncoupler FCCP (Sigma-Aldrich, 25 μM, 2 h) served as positive control. Mitochondrial membrane potential was determined as ratio of red to green fluorescence intensity of the ratiometric mitochondrial dye JC-1. For the determination of the mitochondrial superoxides, the treated cells were loaded with red mitochondrial superoxide indicator (MitoSOX™, 5 µM) in Hanks’ balanced salt solution (HBSS) for 20 min at 37°C. The stained cells were analyzed by using flow cytometry. H_2_O_2_ (50 µM) was used as positive control (El Gaafary et al., 2015).

### Analysis of Cytosolic Reactive Oxygen Species and Caspase 3/7 Activation

Cellular production of reactive oxygen species (ROS) was determined by the cell-permeable fluorescent dye 2′,7′-dichlorodihydrofluorescein diacetate (H_2_DCFDA) (El Gaafary et al., 2015), which upon cleavage of the acetate groups by intracellular esterases and oxidation, is converted to the highly fluorescent 2′,7′-dichlorofluorescein. MDA-MB-231 cells were treated with the most active synthesized benzochromenes (4a, 4c–4f) at 10 μM each for 24 h and then incubated with H_2_DCFDA (10 μM) in HBSS for 20 min at 37°C. The stained cells were analyzed by using flow cytometry. Cells treated with H_2_O_2_ (50 μM) were used as positive control.

For analysis of caspase 3/7 activity, MDA-MB-231 cells were treated with different concentrations of either 4a, 4c–4f, or etoposide for 24 h, and assessed using a Caspase-Glo^®^ 3/7 activity assay (Promega, Madison, WI, USA), which measures caspase 3/7 activity in cell lysates using a luminogenic caspase 3/7 substrate DEVD (Hamed et al., 2020).

### *In vivo* Tumor Model

MDA-MB-231 breast cancer cells were xenotransplanted onto the vascularized but non-innervated chick chorioallantoic membrane (CAM) in medium/Matrigel (1:1, v/v) 8 days after fertilization. The next day, the xenografts were topically treated daily for three consecutive days with 20 μl of benzochromenes 4a, 4c–4f, or etoposide (each at a concentration of 10 µM). Tumor volume was calculated with the formula: V = (L × W × W)/2, where V is the tumor volume, W is the tumor width, and L is the tumor length. On day 12 after fertilization, the xenografts were excised, fixed, and paraffin embedded for histological analysis. Serial sections (5 µm) were deparaffinized before staining with either hematoxylin and eosin (HE) or a specific antibody against the human nuclear proliferation antigen Ki-67 (Glostrup, Denmark). DNA strand breaks in apoptotic cells were visualized by the terminal deoxynucleotidyl transferase dUTP nick-end labeling (TUNEL) method using a TUNEL assay kit (Roche Diagnostics) (El Gaafary et al., 2019; Hamed et al., 2020). The sections were counterstained with hematoxylin and eosin, and images were digitally recorded with an Axio Lab.A1 microscope and a 2/3″ CMOS camera using Progres Gryphax software (all from Carl Zeiss, Oberkochen, Germany). The study protocol complies with the Guide for the Care and Use of Laboratory Animals issued by United States and European regulatory agencies. Avian embryos in this study were sacrificed prior to 7 days before hatching.

### Statistical Analysis

All values are expressed as mean ± standard error of the mean (SEM). Statistical analysis was performed using the Newman–Keuls *post-hoc* test for multigroup comparisons by using SigmaPlot 14.0 (Systat Software Inc., San Jose, CA, USA).

## Results and discussion

### Chemistry

**FIGURE 4:**
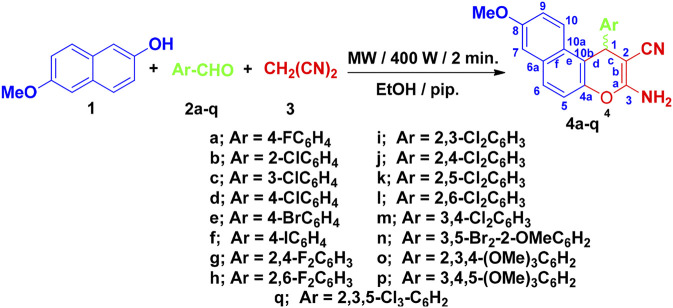
Synthesis of 1H-benzo[f]chromene derivatives 4a–4q.

Here, we have synthesized 17 aryl-substituted 3-amino-1-aryl-8-methoxy-1*H*-benzo[*f*]chromene-2-carbonitriles (4a–4q), which was prepared as depicted in Figure 4. Compounds 4b, 4c, 4f, 4g, 4i, 4l, 4m, and 4o–4q are reported here for the first time. Substituted phenyl at the 1-position of 3-amino-8-methoxy-1*H*-benzo[*f*]chromene-2-carbonitrile (4a–4q) was obtained with high yield by condensation of 6-methoxy-2-naphthol (1) with appropriate aromatic aldehydes (2a–2q) and malononitrile (3) in ethanolic piperidine solution under microwave irradiation at 140 °C for 2 min. The optimum condition was determined to be 400 W and 2 min after yield analysis at different irradiation power (200, 300, 400 W) and time (1, 1.5, 2 min). All the reactions were monitored using TLC technique. It is also important to note that the 1 position of compounds 4a–4q is a chiral center and, as the observed optical rotation of compounds 4a–4q demonstrated, they are optically inactive, which indicates that they are present in the form of racemic (±) mixtures (El-Agrody et al., 2016) as illustrated in Figure 4.

### Spectroscopic Data

The structures and purities of the synthesized compounds 4o–4q were confirmed by spectral analysis. The IR spectra of the newly synthesized compounds 4b, 4c, 4f, 4g, 4i, 4l, 4m, and 4o–4q indicated the presence of the characteristic NH_2_ absorption bands around *υ* 3,476–3,407, *υ* 3,381–3,318, and *υ* 3,291–3,190 cm^−1^ and CN absorption bands around *υ* 2,201–2,183 cm^−1^. On the other hand, the ^1^H-NMR spectra of compounds 4b, 4c, 4f, 4g, 4i, 4l, 4m, and 4o–4q showed the singlet signals of the amino, methine, and methoxy protons in the range of *δ* 7.07–6.93, *δ* 6.09–5.30, and *δ* 3.83–3.82 ppm, respectively. The ^13^C-NMR spectra of compounds 4b, 4c, 4f, 4g, 4i, 4l, 4m, and 4o–4q showed signals resonating in the range of *δ* 55.21–52.59 and *δ* 37.48–28.17 ppm attributable for the methoxy and methine carbons, respectively. Further assignment of the structures of compounds 4b, 4c, 4f, 4g, 4i, 4l, 4m, and 4o–4q was accomplished by mass spectra. The ^13^C-NMR-DEPT for compound 4p and the single crystal x-ray structure analysis of compounds 4h, 4j, 4k, and 4n gave an absolute confirmation for the structures (Amr et al., 2017; Halawa et al., 2017; Okasha et al., 2017; El Gaafary et al., 2021). The ^1^H-NMR and ^13^C-NMR spectra of the synthesized compounds are presented in the Supplementary Data (Supplementary Figures S1–S45). The yield of compounds 4g and 4m was sufficient for spectroscopic analysis, but not for analysis of biological activities. Hence, they were excluded from biological assays.

### Determination of Pharmacokinetic Properties

The oral route of drug administration is still the most frequent and most desired in modern pharmacy. The Lipinski rule implies that ideal drug candidates with good oral bioavailability have the following features: logP ≤5, mw ≤500, number of hydrogen bond acceptors ≤10 (i.e., N or O atoms), and number of hydrogen bond donors ≤5 (Lipinski et al., 2001). Compounds that do not comply with two or more of the rules are not expected to possess sufficient oral bioavailability. Lipinski’s rule of five was used to evaluate drug likeness of the synthesized benzochromene derivatives 4a–4q (Table 1). All benzochromene derivatives except one (4n) exhibited favorable ADME (absorption, distribution, metabolism, and excretion) characteristics indicating that their oral administration would result in high bioavailability and adequate penetration across lipophilic membranes. The compound 4n violated two rules and is, hence, less suitable for oral administration.

**TABLE 1:** Predicted absorption, distribution, metabolism, and excretion (ADME) properties of the synthesized benzochromene derivatives.

Compound	Mw	N-rotb	N-violations	LogS	N-ON	N-OHNH	TPSA	LogP (O/W)	% ABS
4a	346.36	2	0	−4.7530	4	2	68.28	4.10	85.4
4b	362.82	2	0	−5.2268	4	2	68.28	4.56	85.4
4c	362.82	2	0	−5.2577	4	2	68.28	4.59	85.4
4d	362.82	2	0	−5.2783	4	2	68.28	4.61	85.4
4e	407.27	2	0	−5.4122	4	2	68.28	4.74	85.4
4f	454.27	2	1	−5.7006	4	2	68.28	5.02	85.4
4h	364.35	2	0	−4.8148	4	2	68.28	4.16	85.4
4i	397.26	2	1	−5.8757	4	2	68.28	5.19	85.4
4j	397.26	2	1	−5.9066	4	2	68.28	5.22	85.4
4k	397.26	2	1	−5.9066	4	2	68.28	5.22	85.4
4l	397.26	2	1	−5.8757	4	2	68.28	5.19	85.4
4n	516.19	3	2	−6.1538	5	2	77.52	5.46	82.2
4o	418.45	5	0	−4.4028	7	2	77.52	3.76	82.2
4p	418.45	5	0	−4.1968	7	2	95.98	3.56	75.8
4q	431.71	2	1	−6.5246	4	2	68.28	5.82	85.4

Note. Mw, molecular weight; N-Rotb, number of rotatable bonds; LogS, solubility coefficient; N-ON, number of hydrogen bond acceptors; N-OHNH, number of hydrogen bond donors; TPSA, topological polar surface area; LogP, partition coefficient; % ABS, percentage of absorption. Calculations were performed using Molinspiration Cheminformatics (https://www.molinspration.com, Slovensky Grob, Slovakia).

### Analysis of Cytotoxic Activity Against Cancer Cell Lines

The structures of the synthesized 3-amino-1-aryl-8-methoxy-1*H*-benzo[*f*]chromene-2-carbonitriles derivatives (4a–4q) differ in the substitution pattern on the phenyl ring at the 1-position of 1*H*-benzo[*f*]chromene moiety. The phenyl ring could be monohalogenated substituted (4a–4f), dihalogenated substituted (4h–4l), or trisubstituted (4n–4q) with either electron-withdrawing groups like halogens and/or electron-donating groups, like methoxy groups. The antiproliferative and cytotoxic effects of the synthesized benzochromene compounds were further analyzed using cell lines originating from common advanced human cancers. The tested cancer cell lines were the triple-negative breast cancer (TNBC) MDA-MB-231, the non-small cell lung cancer (NSCLC) A549, the cervical cancer HeLa, the pancreatic adenocarcinoma MIA PaCa-2, the urinary bladder carcinoma 5,637, and the hepatocellular carcinoma Hep G2. The clinical anticancer drug etoposide was used as a positive control. Supplementary Figures S46, S47 show concentration-dependent decrease in viable cells after treatment for 24 h with various benzochromene compounds. The respective half-maximal inhibitory concentrations (IC_50_) calculated using the above data are shown in Table 2. As has been previously shown for other benzochromene derivatives with 1*H*-benzo[*f*]chromene scaffold (Kheirollahi et al., 2014; Afifi et al., 2017; Gorle et al., 2017; Ahmed et al., 2018; Fouda et al., 2019; Fouda et al., 2020), most of the synthesized benzochromene compounds were cytotoxic, though to different extents, against the cancer cell lines tested. Interestingly, the synthesized benzochromene derivatives with monohalogenated phenyl ring (4a, 4c–4f), but not 4b, showed the most pronounced cytotoxicity toward all tested cancer cell lines. Their IC_50_ values were in the range of 3.0–12.0 μM. Compound 4c with the 3-monochloro substitution pattern of the pendant phenyl ring (meta position) of the benzo[*f*]chromene scaffold revealed the highest *in vitro* cytotoxicity with IC_50_ range from 3.0 to 6.8 μM toward all the investigated cancer cell lines. Concerning the cytotoxic activity of the synthesized benzochromene derivatives with dihalogenated phenyl ring, compounds 4i–4k exhibited higher cytotoxic activity than compounds 4h and 4l. Finally, we demonstrated that among the benzochromene derivatives with trisubstituted phenyl ring, compounds 4o and 4p showed weaker cytotoxic activity than compounds 4n and 4q. Thus, compounds 4b, 4h, 4o, and, except for one cancer cell line, 4p exhibited rather low toxicity against cancer cells lines with IC_50_ >30 μM.

**TABLE 2:** Cytotoxicity of benzochromene derivatives against a panel of solid cancer cell lines.

Compound		Cell lines, IC_50_ (µM), 24 h 0 30					
		MDA-MB-231	A549	HeLa	MIA PaCa-2	5,367	Hep G2
Monosubstituted	4a	8.9 ± 1.3	7.9 ± 0.5	9.1 ± 1.1	6.9 ± 0.2	7.3 ± 0.5	8.2 ± 0.3
	4b	>30.0	>30.0	>30.0	>30.0	>30.0	>30.0
	4c	5.9 ± 0.6	5.6 ± 0.3	5.6 ± 1.0	5.2 ± 0.6	3.0 ± 0.5	6.8 ± 0.1
	4d	6.4 ± 1.5	6.8 ± 0.2	7.7 ± 0.7	6.7 ± 0.5	7.1 ± 0.5	7.0 ± 0.1
	4e	6.8 ± 0.3	6.9 ± 0.2	7.4 ± 0.5	6.2 ± 0.3	6.6 ± 0.5	7.1 ± 0.1
	4f	8.1 ± 0.2	9.8 ± 1.1	12.0 ± 1.8	7.9 ± 0.6	6.7 ± 0.6	7.8 ± 0.2
Bisubstituted	4h	>30.0	>30.0	>30.0	≥30.0	>30.0	>30.0
	4i	16.0 ± 1.6	9.4 ± 0.6	20.8 ± 2.7	8.5 ± 0.3	8.6 ± 1.3	21.1 ± 3.3
	4j	15.3 ± 3.5	11.3 ± 2.2	15.2 ± 2.4	7.5 ± 0.3	8.0 ± 0.6	14.4 ± 3
	4k	10.7 ± 1.5^a^	7.7 ± 0.7^a^	19.8 ± 2.2^a^	7.3 ± 0.7	6.5 ± 1.6	18.6 ± 5.9
	4l	20.3 ± 4.3	20.4 ± 2.4	23.4 ± 1.7	19.5 ± 5.0	14.1 ± 1.2	18.7 ± 3.4
Trisubstituted	4n	12.4 ± 3.5	12.0 ± 2.0	10.7 ± 1.9	6.7 ± 0.6	5.7 ± 0.3	9.2 ± 0.4
	4o	>30.0	>30.0	>30.0	>30.0	>30.0	>30.0
	4p	>30.0	>30.0	>30.0	>30.0	14.0 ± 7.9	>30.0
	4q	11.6 ± 0.9	9.3 ± 1.2	21.3 ± 2.2	9.5 ± 0.6	7.3 ± 0.2	19.2 ± 3.6
	Etoposide	>30.0^a^	>30.0^a^	15.0 ± 4.5^a^	>30.0	N.D	N.D

Note. N.D, not done; IC_50_, half maximal inhibitory concentration.

a (El Gaafary et al., 2021).

The cytotoxic effects of benzochromene derivatives on cancer cell lines were further normalized to respective cell line doubling time and are presented as GR_50_. Different from IC_50_ values, GR_50_ is the cell division rate and, hence, assay duration independent (Hafner et al., 2016). Efficacy (GR_max_) indicates the maximal drug response on growth rate, and it scores between −1 and 1. A value of 0 corresponds to a fully cytostatic response, whereas positive and negative values indicate partial inhibition or partial cytotoxic response, respectively. The normalized growth rate inhibition metrics, GR_50_ and GR_max_ values of the synthesized 1*H*-benzo[*f*]chromene derivatives (4a–4q), are presented in Table 3. Using these metrics, we found that the synthesized benzochromene derivatives with monohalogenated phenyl ring (4a, 4c–4f) had the most pronounced cytotoxicity toward all tested cancer cell lines with a GR_50_ range of 1.0–8.1 μM and GR_max_ range of −0.76–−1, and compound 4c showed the greatest *in vitro* antiproliferative/cytotoxic activities against the six different types of cancer cell lines with a GR_50_ range of 1.0–4.9 μM and GR_max_ range of −0.9–−1. These data obtained from the normalized growth rate inhibition metrics were in accordance with those obtained from IC_50_ metrics, indicating the accuracy of both parameters in the determination of drug potency.

**TABLE 3:** Inhibitory effect of benzochromene derivatives on growth of a panel of solid cancer cell lines.

			Cell lines, 24 h					
			MDA-MB-231	A549	HeLa	MIA PaCa-2	5,367	Hep G2
Doubling time (h)			28	24	24	36	24	24
Monosubstituted	4a	GR_50,_ µM	6.6	6.6	7.0	3.9	5.3	6.5
		GR_max_	−0.99	−0.99	−0.99	−1	−0.98	−0.97
	4b	GR_50,_ µM	8.5	13.6	12.3	12.2	14.1	29.9
		GR_max_	0.05	0.27	0.38	−0.07	0.18	0.49
	4c	GR_50,_ µM	3.0	2.9	2.2	2.0	1.0	4.9
		GR_max_	−1	−1	−0.90	−1	−1	−0.98
	4d	GR_50,_ µM	5.7	2.7	6.1	4.8	5.4	5.1
		GR_max_	−0.99	−1	−0.94	−1	−1	−0.98
	4e	GR_50,_ µM	4.7	5.1	5.5	3.5	4.7	5.0
		GR_max_	−1	−1	−0.86	−1	−1	−1
	4f	GR_50,_ µM	4.9	7.6	8.1	5.6	5.1	6.3
		GR_max_	−0.98	−0.96	−0.76	−1	−1	−0.96
Bisubstituted	4h	GR_50,_ µM	5.6	13.9	20.0	12.0	18.1	12.7
		GR_max_	0.04	0.14	0.30	−0.29	0.32	0.06
	4i	GR_50,_ µM	6.7	5.6	9.2	4.0	5.0	9.5
		GR_max_	−0.78	−0.82	−0.32	−0.93	−0.86	−0.38
	4j	GR_50,_ µM	8.8	8.0	8.5	4.3	6.6	8.5
		GR_max_	−0.76	−0.86	−0.54	−0.98	−0.94	−0.58
	4k	GR_50,_ µM	3.3	4.0	8.7	3.2	3.6	8.0
		GR_max_	−0.85	−0.86	−0.32	−0.94	−0.94	−0.58
	4l	GR_50,_ µM	6.5	8.5	11.6	7.8	6.3	8.8
		GR_max_	−0.45	−0.38	−0.34	−0.64	−0.74	−0.48
Trisubstituted	4n	GR_50,_ µM	3.3	5.7	6.7	2.9	3.1	7.5
		GR_max_	−0.80	−0.64	−0.64	−0.98	−0.98	−0.80
	4o	GR_50,_ µM	17.1	Inf.	22.4	6.4	9.7	Inf.
		GR_max_	0.39	0.52	0.46	−0.02	0.06	0.96
	4p	GR_50,_ µM	6.2	6.2	7.9	0.3	1.9	Inf.
		GR_max_	0.02	0.42	0.12	0.27	−0.30	0.58
	4q	GR_50,_ µM	4.9	5.3	9.8	0.9	3.7	10.0
		GR_max_	−0.92	−0.88	−0.36	−0.85	−0.80	−0.51

Note. GR_50_, half maximal growth rate inhibition; GR_max_, maximal growth rate inhibition; Inf., infinite.

The difference in cytotoxic activity of the synthesized benzochromene derivatives with different substitution patterns at the phenyl ring prompted us to further study the mechanisms that impede proliferation and induce cell death.

### Effect of Benzochromene Derivatives on Cell Cycle Progression of Breast Cancer Cells

**FIGURE 5:**
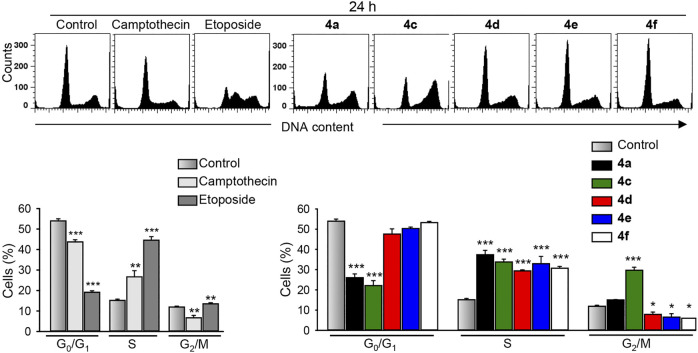
The benzochromene derivatives 4a, 4c–4f monohalogenated at phenyl ring induce cell cycle perturbations in breast cancer cells. MDA-MB-231 cells were treated with either benzochromene derivatives, camptothecin, or etoposide, each 10 μM, for 24 h, stained with propidium iodide, and analyzed by flow cytometry. Representative histograms are shown. Data are mean ± SEM, *n* = 3, *p < 0.05, **p < 0.01, ***p < 0.001 vs. control.

Cell cycle analyses (Figure 5) revealed that treatment of MDA-MB-231 cells with the most active benzochromene derivatives (4a, 4c–4f) at a concentration of 10 μM for 24 h resulted in a significant accumulation of the MDA-MB-231 cells in the S phase of the cell cycle. A concentration of 10 μM was selected based on the IC_50_ values of tested compounds. The same concentration was used for all compounds to enable their comparison. Compound 4c with the highest cytotoxic activity was the only compound that induced, in addition, a strong increase in cell number in the G_2_/M phase. The positive controls, etoposide, and camptothecin, at this time point mostly increased the number of cells in the S phase of the cell cycle.

### Molecular Mechanisms of Cell Death

**FIGURE 6:**
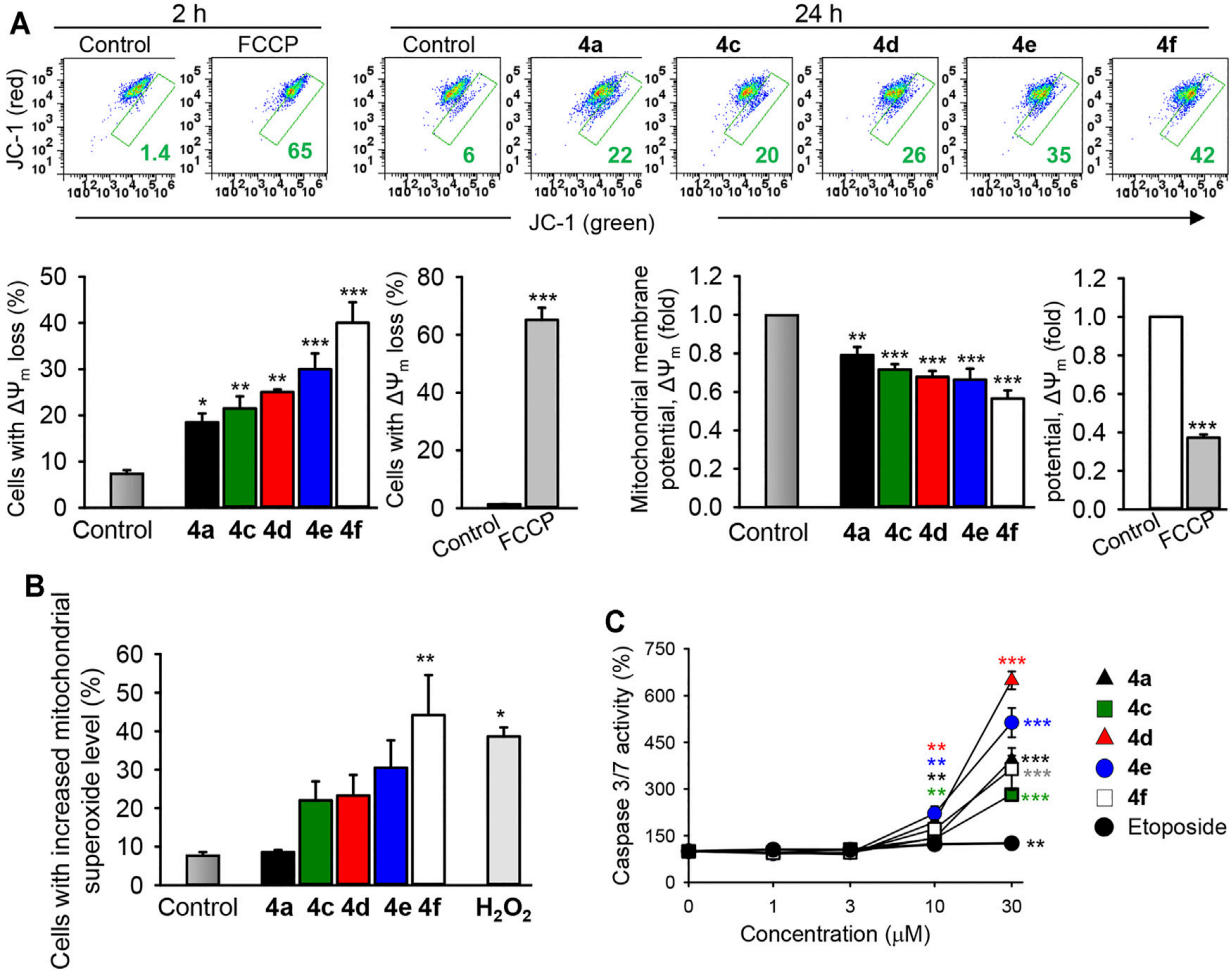
The benzochromene derivatives monohalogenated at the phenyl ring affect the mitochondrial integrity, increase mitochondrial superoxide anions, and induce the activation of caspase 3/7 in breast cancer cell lines. **(A)** MDA-MB-231 cells were treated with the most active benzochromene derivatives at a concentration of 10 μM for 24 h or the respiratory uncoupler, FCCP (25 μM), used as a positive control, for 2 h. The mitochondrial membrane potential was analyzed flow cytometrically by using the JC-1 dye. Representative dot plots are shown. Numbers next to all gates represent the percentages of cells with the loss of mitochondrial membrane potential (ΔΨ_m_). Graphs show the percentages of MDA-MB-231 cells with depolarized mitochondria and the degree of ΔΨ_m_ loss in the treated cells. **(B)** The cells were treated as in **(A)**, stained with MitoSOX™ red mitochondrial superoxide indicator, and analyzed by flow cytometry. H_2_O_2_ (50 μM), positive control. **(C)** The activity of caspase 3/7 in cell lysates was assessed using a luminometric substrate. All data are mean ± SEM, *n* = 3, *p < 0.05, **p < 0.01, ***p < 0.001 vs. control.

The mitochondria is a critical regulator of many cellular processes such as cell death, cell signaling circuitry, and energetic homeostasis. The mitochondria clearly have essential roles in the apoptotic cell death by integrating and propagating cell death signals originating from both the extrinsic and intrinsic apoptotic signaling pathways (Tait & Green, 2012). To delineate the importance of the mitochondria in the induction of cancer cell death by the most active benzochromene derivatives (4a, 4c–4f), the mitochondrial membrane integrity was analyzed by using the lipophilic cationic JC-1 dye that displays membrane potential-dependent accumulation in the mitochondria. Inside the intact mitochondria, the dye forms red fluorescent aggregates, while in the cells with damaged mitochondria, the dye regains its green fluorescence that can be detected by using flow cytometry. Interestingly, all the analyzed synthesized benzochromene derivatives, at a concentration of 10 μM, induced a pronounced collapse of mitochondrial membrane potential in TNBC MDA-MB-231 cells after 24 h (Figure 6A), with the compound 4f displaying the highest activity. The percentage of cancer cells with deenergized mitochondria was increased to about 18%–39% for 4a and 4c–4f compared with 5% in the untreated control cells.

The mitochondrial membrane potential is controlled by electron flux through the respiratory chain and proton circuit across the mitochondrial inner membrane. Electrons leaked in the mitochondria are a major source for superoxide anions, which might be converted to other types of reactive oxygen radicals (ROS) leading to the initiation of cellular oxidative damage and cancer cell death (Ricci et al., 2003). Indeed, in accordance with the percentages of cells with damaged mitochondria, treatment of MDA-MB-231 cells with 4f (10 μM) significantly increased the percentages of cells exhibiting elevated mitochondrial superoxide anions to 44%, relative to the control (7.8%) (Figure 6B), supporting its mitochondrial-targeting activity.

The predominant form of programmed cell death is apoptosis, a process that requires mostly the activation of the caspase proteases (Wang & Youle, 2009). Caspases 3 and 7 are the best characterized executioners of the apoptotic cascade, which, when activated, cleave a large set of substrates leading to rapid apoptotic cell death featuring a characteristic set of morphological and biochemical changes (Fink & Cookson, 2005). Treatment of the TNBC MDA-MB-231 cells with different concentrations of the benzochromene derivatives with a monohalogenated phenyl ring (4a, 4c–4f) for 24 h induced a strong caspase 3/7 activation in a concentration-dependent manner, although in varying degrees (Figure 6C). Compounds 4d and 4e increased the activity of caspase 3/7 more strongly than compounds 4a, 4c, and 4f. Compared with the tested benzochromene derivatives, the chemotherapeutic drug etoposide showed a moderate effect on the activation of caspase 3/7 in the MDA-MB-231 cells.

**FIGURE 7:**
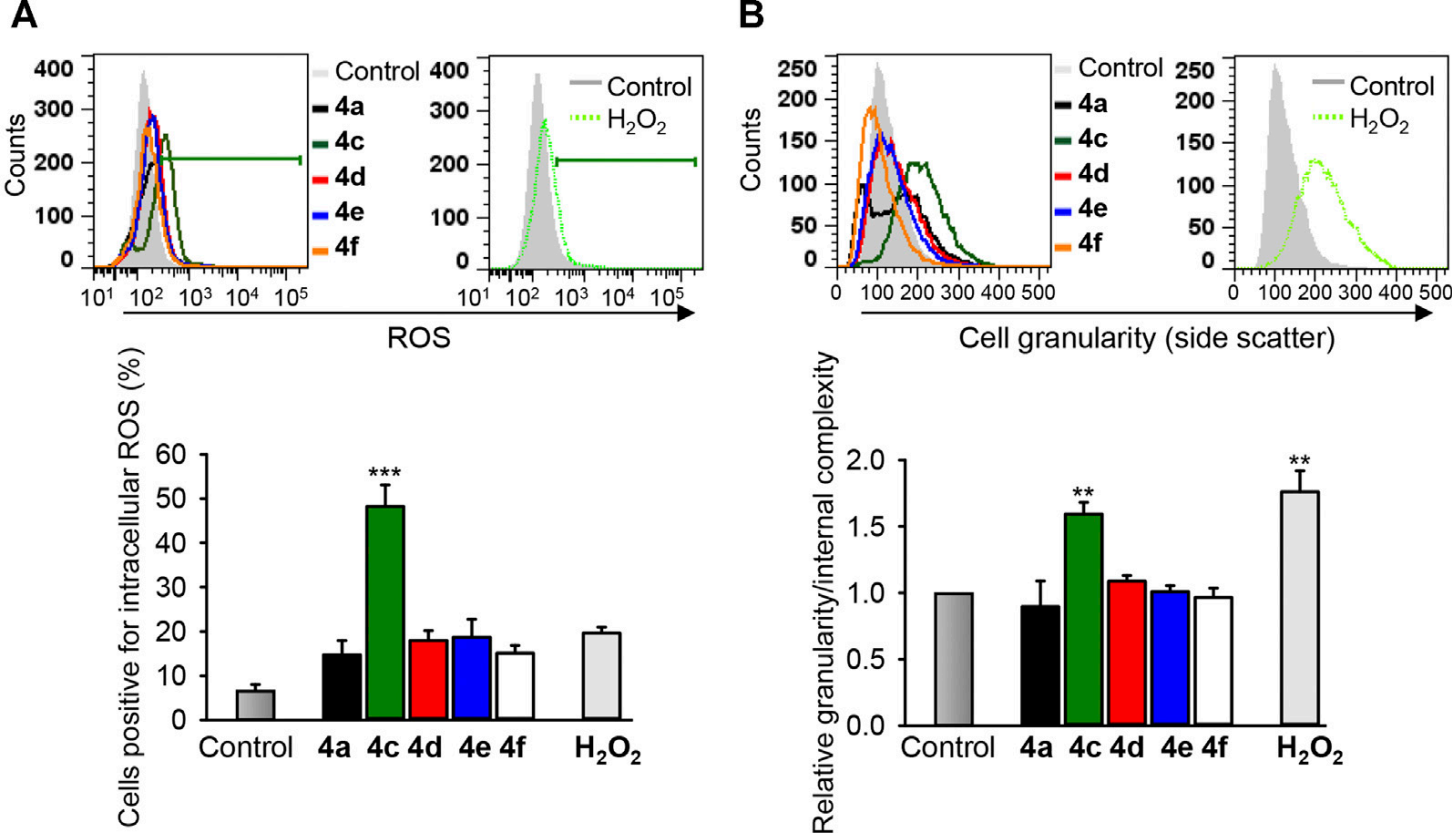
The benzochromene derivative 4c increases cytosolic ROS and granularity/internal complexity in the triple-negative breast cancer cells. MDA-MB-231 cells were treated with 10 μM of benzochromene derivatives 4a, 4c–4f for 24 h. **(A)** The treated cells were stained with reactive oxygen species (ROS) indicator H_2_DCFDA and analyzed by flow cytometry. **(B)** Cells were treated as in **(A)** and analyzed by flow cytometry. Increase in side scatter intensity is shown. Representative histograms are shown. Graphs show the increase in intracellular ROS and cell granularity/internal complexity. All data are mean ± SEM, *n* = 3, **p < 0.01, ***p < 0.001 vs. control.

In cytotoxicity analysis, which provides, in the form of IC_50_ values, suitable for comparison between different compounds, compound 4c showed the highest cytotoxic activity against all cancer cell lines tested compared with other benzochromene derivatives, but this compound had less pronounced effects on mitochondrial membrane integrity or on caspase 3/7 activation in cancer cells. Intriguingly, among the active benzochromene derivatives with a monohalogenated phenyl ring (4a, 4c–4f), only compound 4c induced a pronounced increase in cytosolic ROS level (Figure 7). ROS are toxic molecules that cause oxidative damage to cellular components like lipids, proteins, and DNA, leading to deterioration of cellular functions and subsequent cell death (Trachootham et al., 2009). The ROS-induced cell death could be caspase-dependent or -independent (Holze et al., 2018). On the other hand, ROS generation may be a relatively late event during cell death, occurring after cells have started on a process of caspase activation (Ricci et al., 2003). In addition to the increase in cytosolic ROS, compound 4c significantly increased the cell granularity/internal complexity of the treated MDA-MB-231 cells (Figure 7B), indicating its potency in the induction of cell death.

### Analysis of Antitumor Efficacy of Benzochromene Derivatives *in vivo*

**FIGURE 8:**
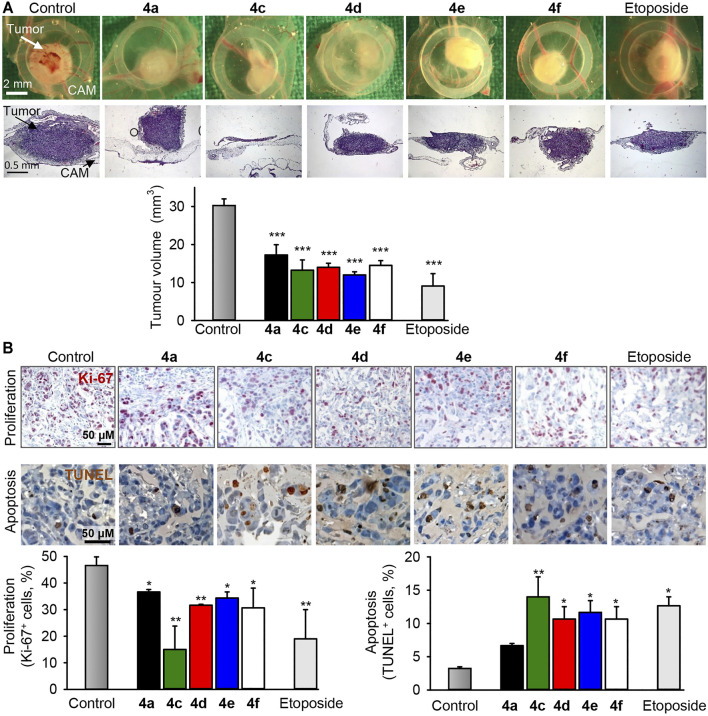
The benzochromene derivatives 4a, 4c–4f inhibit growth and induce apoptosis in preestablished triple-negative breast cancer xenografts *in vivo*. MDA-MB-231 cells were grafted onto the chorioallantoic membrane (CAM) of fertilized chick eggs. The next day, the tumors were topically treated daily for 3 days with compounds (4a, 4c–4f, 10 μM each) or etoposide (10 μM). **(A)** Representative pictures of tumor xenografts immediately after extraction (first row) and hematoxylin and eosin (HE, second row) staining of tumors (original magnification ×50). Graph shows the calculated tumor volume. **(B)** Immunohistochemical analysis of tumor cell proliferation using an antibody against the Ki-67 nuclear antigen (proliferation marker, dark violet stain within nuclei, first row) and TUNEL staining (TdT, dark brown, second row) for the detection of apoptosis. Original magnification ×200. Graphs show the quantification of tumor cell proliferation and the quantification of apoptotic tumor cells, *n* = 3–4, *p < 0.05, **p < 0.01 vs. control.

Breast cancer xenografts were preestablished by seeding TNBC MDA-MB-231 cells on the chorioallantoic membranes of fertilized chick eggs (CAM). All compounds tested (4a, 4c–4f) induced reduction of tumors at least by half (Figure 8A), which was comparable with the effect of etoposide, a drug used in the clinic to treat breast cancer. Immunohistochemical analysis of the treated MDA-MB-231 xenografts demonstrated that the benzochromene derivatives with monohalogenated phenyl ring (4a, 4c–4f) reduced proliferation of cancer cells, as can be determined by the diminished expression of the nuclear antigen Ki-67. In addition, all compounds induced apoptosis as can be determined by an increase in the DNA strand breaks, which is a typical sign for apoptosis (Figure 8B). In this assay, in agreement with *in vitro* cytotoxicity analysis, compound 4c exhibited the highest potency among the benzochromene derivatives tested. Importantly, benzochromene derivatives induced neither death of chicken embryos nor any other visual macroscopic signs of systemic toxicity indicating a low level of undesirable side effects of the compounds tested.

### Structure–Activity Relationship Analysis

The structure–activity relationship (SAR) study revealed several crucial structural requirements, which enhanced the potency of the *β*-enaminonitrile linked to 8-methoxy-1*H*-benzo[*f*]chromenes. The variations in the substitution pattern, including the type of substituents (electron-donating groups, electron-withdrawing groups, or both of them) and their position on the phenyl ring at the 1-position of 1*H*-benzo[*f*]chromene moiety, affected the cytotoxic activity of the synthesized benzochromene derivatives toward the tested cancer cell lines. Interestingly, we observed that the grafting of one lipophilic electron-withdrawing group, such as a halogen at either the meta or the para position of the phenyl ring at the 1-position of 1*H*-benzo[*f*]chromene moiety, led to a pronounced cytotoxic activity toward all tested cancer cell lines. However, the presence of the lipophilic electron-withdrawing group at the ortho position did not result in any significant cytotoxic activity at the tested concentration range. More interestingly, we noticed a difference in cell death mechanisms when the grafting of the halogen atom of moderate size occurs at either the meta or the para position of the phenyl ring. Among the benzochromene compounds with monohalogenated phenyl ring, only compound 4c has a chlorine atom at the meta position of the phenyl ring, and its mechanism of action in inhibition of the proliferation and induction of death seems to be different from that of compounds 4d–4f, which show halogen atoms of moderate size, like chlorine, bromine, and iodine at the para position of the phenyl ring. Only compound 4c accumulated the treated MDA-MB-231 cells in the S and G_2_/M phases of the cell cycle, induced ROS, and increased cell granularity/internal complexity. In addition, its effect on mitochondrial depolarization and caspase 3/7 activation was less pronounced than that of compounds 4d–4f, which induced accumulation of cancer cells only in the S phase of the cell cycle. Among benzochromene compounds with a para-monohalogenated phenyl ring, compound 4f with para-monoiodinated phenyl ring had the strongest effect on mitochondrial integrity and membrane potential, as well as on mitochondrial superoxide anions, indicating its mitochondrial-targeting ability, while compounds 4c and 4d with a para-monochlorinated and a para-monobrominated phenyl ring, respectively, had the strongest effect on the caspase 3/7 activity, and their effect on mitochondrial integrity and membrane potential was lower than that of compound 4f indicating that a death receptor signaling pathway might be involved.

In the benzochromene compounds with dihalogenated phenyl ring, the presence of two electron-withdrawing groups, with one of them at the ortho position, decreased their cytotoxic activity in comparison with the benzochromene compounds with monohalogenated phenyl ring with an electron-withdrawing group at either the meta or the para position. In addition, the presence of lipophilic electron-donating substituents like methoxy groups in compounds with trisubstituted phenyl ring decreased pronouncedly the cytotoxic activity of benzochromene compounds toward all the tested cell lines. This decrease in the activity occurred independently of the position of substituents on the benzene ring. We could hypothesize that the presence of either lipophilic electron-donating substituents at any position of the phenyl ring or electron withdrawing group at the ortho position in combination with two electron withdrawing groups at either the meta or the para position would decrease the cytotoxic activity of the benzochromene compounds, in comparison with the benzochromene compounds with dihalogenated or trisubstituted phenyl ring with electron-withdrawing groups at either the meta or the para position.

In summary, 17 aryl-substituted 3-amino-1-aryl-8-methoxy-1*H*-benzo[*f*]chromene-2-carbonitriles (4a–4q) were synthesized and evaluated for their cytotoxic and apoptosis-inducing activity. Benzochromene compounds with a monohalogenated phenyl ring (4a, 4c–4f) exhibited the most pronounced cytotoxicity, with 4c displaying the highest cytotoxic activity *in vitro* and *in vivo*. Compound 4c with one chlorine atom at the meta position of the phenyl ring induced accumulation of breast cancer cells in the S and G_2_/M phases of the cell cycle, induced ROS, and increased cell granularity/internal complexity. Differently, compounds 4d–4f with halogen atoms at the para position of the phenyl ring were stronger inducers of mitochondrial membrane depolarization and caspase 3/7 activation compared with 4c. Compounds 4d–4f induced accumulation of breast cancer cells only in the S phase of the cell cycle, and compound 4f had the strongest effect on mitochondrial integrity and membrane potential dissipation, as well as on the induction of mitochondrial superoxide anions, indicating its mitochondrial-targeting ability. The structure–activity relationship (SAR) study of the synthesized benzochromene compounds confirmed that the presence of lipophilic electron-withdrawing group, like halogens at meta or para positions of the phenyl ring at the 1 position of 1*H*-benzo[*f*]chromene nucleus, is responsible for the enhancement of cytotoxic activity against different cancer cell lines and define the molecular mechanism involved in cell death.

## Data Availability

The original contributions presented in the study are included in the article/Supplementary Material, further inquiries can be directed to the corresponding authors.
