# Supplementary material for: Synthesis of β-Enaminonitrile-Linked 8-Methoxy-1H-Benzo[f]Chromene Moieties and Analysis of Their Antitumor Mechanisms
Source: Front Chem. 2021 Nov 22;9:759148. doi: 10.3389/fchem.2021.759148 (PMC8645891; doi:10.3389/fchem.2021.759148)
Supplement: Supplementary file 1 [file DataSheet1.docx]

***Supplementary Material***

Synthesis of β-Enamionitriles Linked 8-Methoxy-1*H*-benzo[*f*]chromene Moieties and Analysis of Their Antitumor Mechanisms

**Menna Elgaafary^1,2^, Ahmed M. Fouda^3^, Hany M. Mohamed^4,5^, Abdelaaty Hamed^5^, Heba K. A. El-Mawgoud^6^, Lu Jin^1^, Judith Ulrich^1^, Thomas Simmet^1^, Tatiana Syrovets^1,^*^, †^, Ahmed M. El-Agrody^5,^*^, †^**

| **Table of contents** | **Page** |
| --- | --- |
| **Figure S1.** ^1^H-NMR spectrum (DMSO-*d_6_*, 500 MHz) of compound **4a**. | S4 |
| **Figure S2.** Enlarged ^1^H-NMR spectrum (DMSO-*d_6_*, 500 MHz) of compound **4a**. | S5 |
| **Figure S3.** ^13^C-NMR spectrum (DMSO-*d_6_*, 125 MHz) of compound **4a**. | S6 |
| **Figure S4.** ^1^H-NMR spectrum (DMSO-*d_6_*, 500 MHz) of compound **4b**. | S7 |
| **Figure S5.** Enlarged ^1^H-NMR spectrum (DMSO-*d_6_*, 500 MHz) of compound **4b**. | S8 |
| **Figure S6.** ^13^C-NMR spectrum (DMSO-*d_6_*, 125 MHz) of compound **4b**. | S9 |
| **Figure S7.** ^1^H-NMR spectrum (DMSO-*d_6_*, 500 MHz) of compound **4c**. | S10 |
| **Figure S8.** Enlarged ^1^H-NMR spectrum (DMSO-*d_6_*, 500 MHz) of compound **4c**. | S11 |
| **Figure S9.** ^13^C-NMR spectrum (DMSO-*d_6_*, 125 MHz) of compound **4c**. | S12 |
| **Figure S10.** ^1^H-NMR spectrum (DMSO-*d_6_*, 500 MHz) of compound **4d**. | S13 |
| **Figure S11.** Enlarged ^1^H-NMR spectrum (DMSO-*d_6_*, 500 MHz) of compound **4d**. | S14 |
| **Figure S12.** ^13^C-NMR spectrum (DMSO-*d_6_*, 125 MHz) of compound **4d**. | S15 |
| **Figure S13.** ^1^H-NMR spectrum (DMSO-*d_6_*, 500 MHz) of compound **4e**. | S16 |
| **Figure S14.** Enlarged ^1^H-NMR spectrum (DMSO-*d_6_*, 500 MHz) of compound **4e**. | S17 |
| **Figure S15.** ^13^C-NMR spectrum (DMSO-*d_6_*, 125 MHz) of compound **4e**. | S18 |
| **Figure S16.** ^1^H-NMR spectrum (DMSO-*d_6_*, 500 MHz) of compound **4f**. | S19 |
| **Figure S17.** Enlarged ^1^H-NMR spectrum (DMSO-*d_6_*, 500 MHz) of compound **4f**. | S20 |
| **Figure S18.** ^13^C-NMR spectrum (DMSO-*d_6_*, 125 MHz) of compound **4f**. | S21 |
| **Figure S19.** ^1^H-NMR spectrum (DMSO-*d_6_*, 500 MHz) of compound **4g**. | S22 |
| **Figure S20.** Enlarged ^1^H-NMR spectrum (DMSO-*d_6_*, 500 MHz) of compound **4g**. | S23 |
| **Figure S21.** ^13^C-NMR spectrum (DMSO-*d_6_*, 125 MHz) of compound **4g**. | S24 |
| **Figure S22.** ^1^H-NMR spectrum (DMSO-*d_6_*, 500 MHz) of compound **4h**. | S25 |
| **Figure S23.**  Enlarged ^1^H-NMR spectrum (DMSO-*d_6_*, 500 MHz) of compound **4h**. | S26 |
| **Figure S24.** ^13^C-NMR spectrum (DMSO-*d_6_*, 500 MHz) of compound **4h**. | S27 |
| **Figure S25.** ^1^H-NMR spectrum (DMSO-*d_6_*, 500 MHz) of compound **4i**. | S28 |
| **Figure S26.** Enlarged ^1^H-NMR spectrum (DMSO-*d_6_*, 500 MHz) of compound **4i**. | S29 |
| **Figure S27.** ^13^C-NMR spectrum (DMSO-*d_6_*, 125 MHz) of compound **4i**. | S30 |
| **Figure S28.** ^1^H-NMR spectrum (DMSO-*d_6_*, 500 MHz) of compound **4k**. | S31 |
| **Figure S29.** Enlarged ^1^H-NMR spectrum (DMSO-*d_6_*, 500 MHz) of compound **4k**. | S32 |
| **Figure S30.** ^13^C-NMR spectrum (DMSO-*d_6_*, 125 MHz) of compound **4k**. | S33 |
| **Figure S31**. ^1^H-NMR spectrum (DMSO-*d_6_*, 500 MHz) of compound **4l**. | S34 |
| **Figure S32.** Enlarged ^1^H-NMR spectrum (DMSO-*d_6_*, 500 MHz) of compound **4l**. | S35 |
| **Figure S33.** ^13^C-NMR spectrum (DMSO-*d_6_*, 125 MHz) of compound **4l**. | S36 |
| **Figure S34.** ^1^H-NMR spectrum (DMSO-*d_6_*, 500 MHz) of compound **4m**. | S37 |
| **Figure S35.** Enlarged ^1^H-NMR spectrum (DMSO-*d_6_*, 500 MHz) of compound **4m**. | S38 |
| **Figure S36.** ^13^C-NMR spectrum (DMSO-*d_6_*, 125 MHz) of compound **4m**. | S39 |
| **Figure S37.** ^1^H-NMR spectrum (DMSO-*d_6_*, 500 MHz) of compound **4n**. | S40 |
| **Figure S38.** Enlarged ^1^H-NMR spectrum (DMSO-*d_6_*, 500 MHz) of compound **4n**. | S41 |
| **Figure S39.** ^13^C-NMR spectrum (DMSO-*d_6_*, 125 MHz) of compound **4n**. | S42 |
| **Figure S40.** ^1^H-NMR spectrum (DMSO-*d_6_*, 500 MHz) of compound **4p**. | S43 |
| **Figure S41.** ^13^C-NMR spectrum (DMSO-*d_6_*, 125 MHz) of compound **4p**. | S44 |
| **Figure S42.** DEPT spectrum (DMSO-*d_6_*, 125 MHz) of compound **4p**. | S45 |
| **Figure S43.** ^1^H-NMR spectrum (DMSO-*d_6_*, 500 MHz) of compound **4q**. | S46 |
| **Figure S44.** ^13^C-NMR spectrum (DMSO-*d_6_*, 125 MHz) of compound **4q**. | S47 |
| **Figure S45.** APT spectrum (DMSO-*d_6_*, 125 MHz) of compound **4q**.  **Figure S46.** Cytotoxic activity of synthesized benzochromene derivatives against cancer cell lines MDA-MB-231, A549, and HeLa.  **Figure S47.** Cytotoxic activity of synthesized benzochromene derivatives against cancer cell lines MIA PaCa-2, 5367, and Hep G2. | S48  S49  S50 |


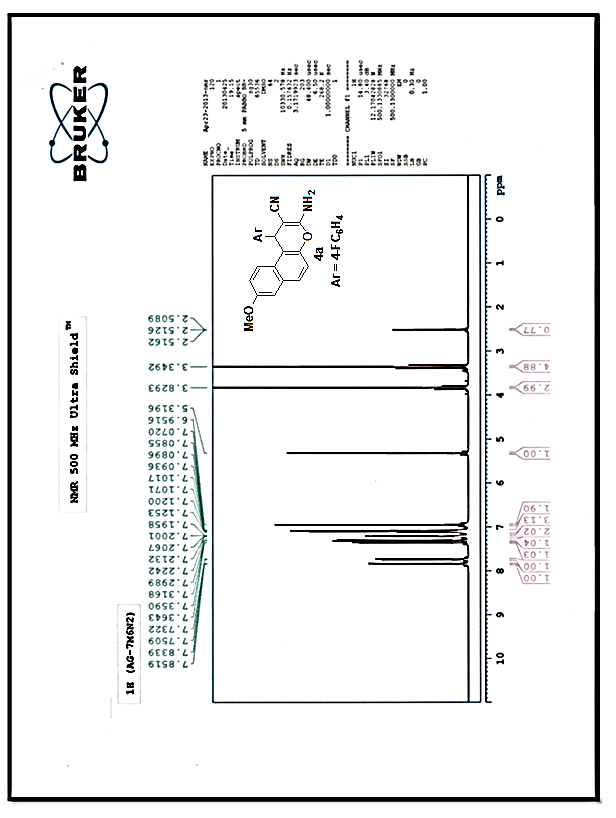


**Figure S1**. ^1^H-NMR spectrum (DMSO-*d_6_*, 500 MHz) of compound **4a.**


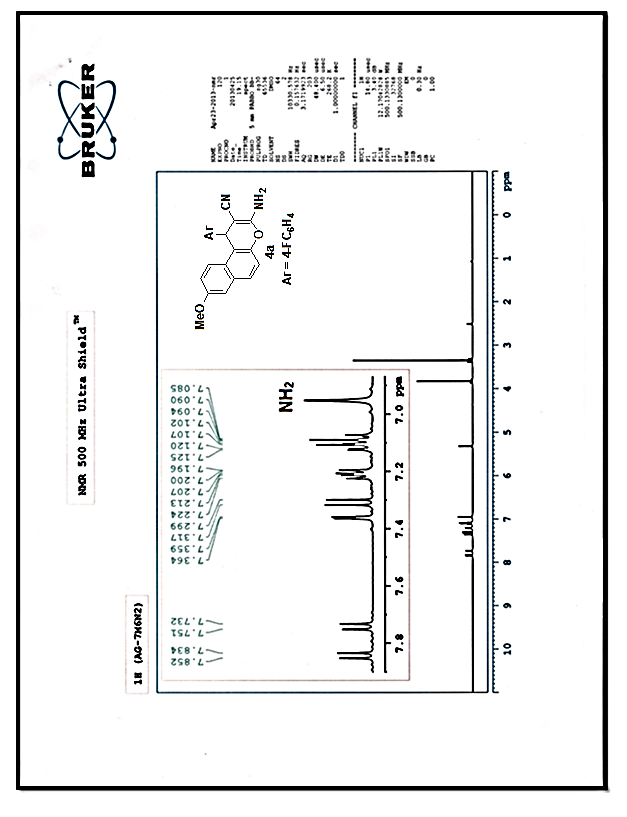


**Figure S2.** Enlarged ^1^H-NMR spectrum (DMSO-*d_6_*, 500 MHz) of compound **4a.**


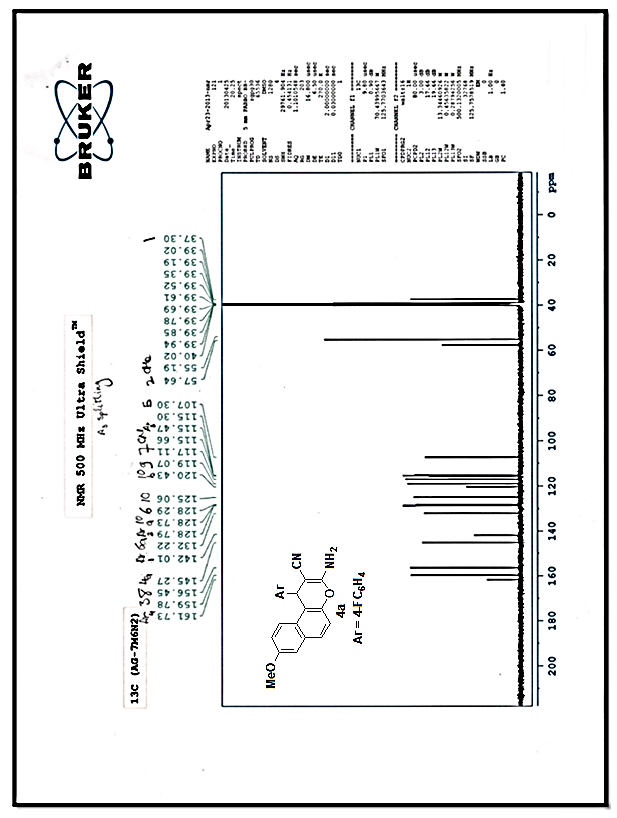


**Figure S3.** ^13^C-NMR spectrum (DMSO-*d_6_*, 125 MHz) of compound **4a.**


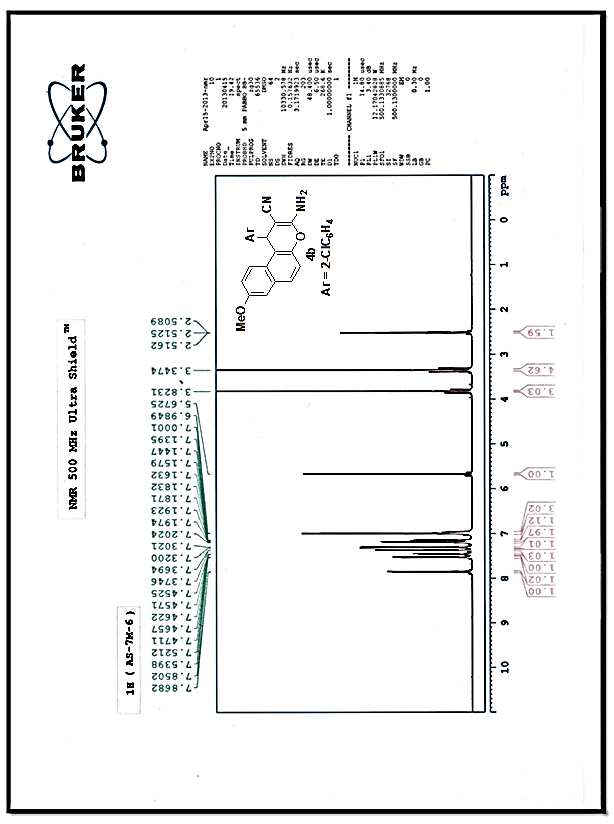


**Figure S4.** ^1^H-NMR spectrum (DMSO-*d_6_*, 500 MHz) of compound **4b.**


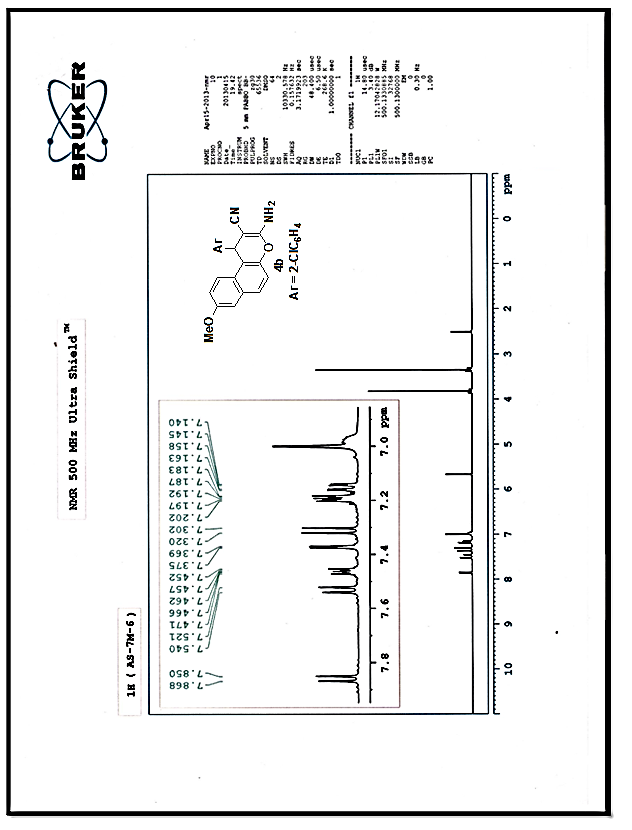


**Figure S5.** Enlarged ^1^H-NMR spectrum (DMSO-*d_6_*, 500 MHz) of compound **4b.**


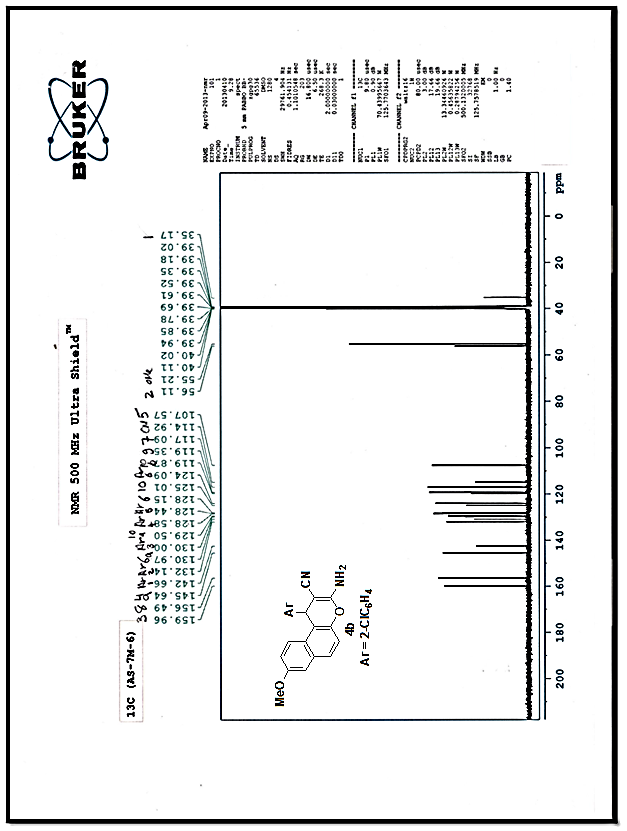


**Figure S6.** ^13^C-NMR spectrum (DMSO-*d_6_*, 125 MHz) of compound **4b.**


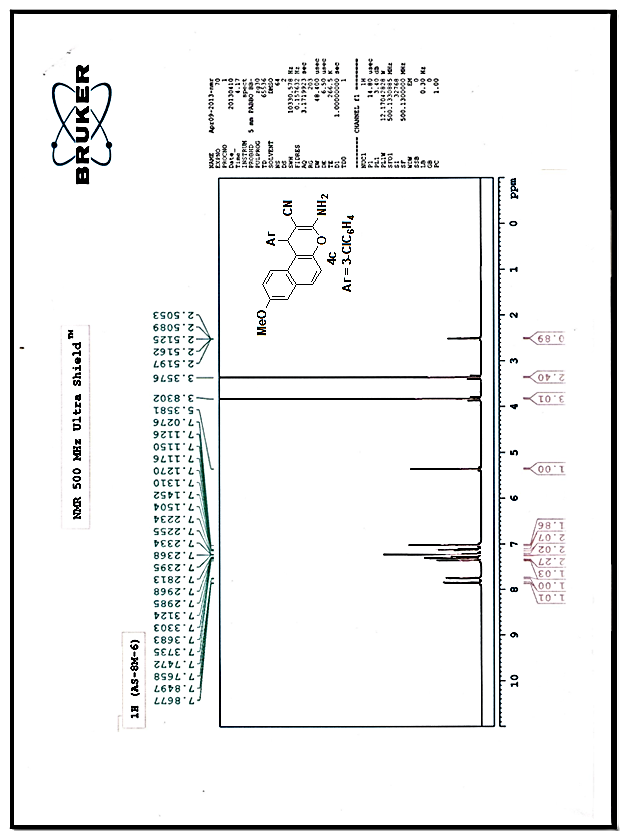


**Figure S7.** ^1^H-NMR spectrum (DMSO-*d_6_*, 500 MHz) of compound **4c.**


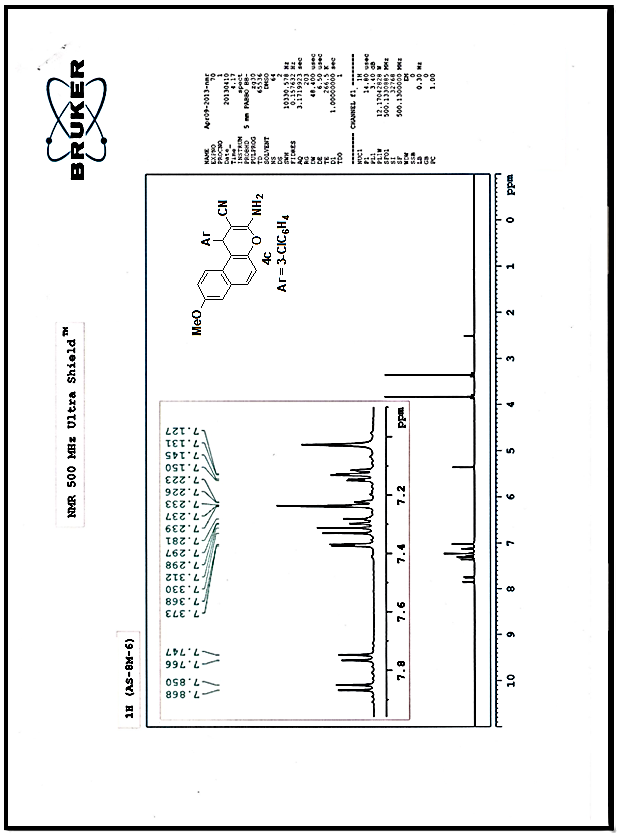


**Figure S8.** Enlarged ^1^H-NMR spectrum (DMSO-*d_6_*, 500 MHz) of compound **4c.**


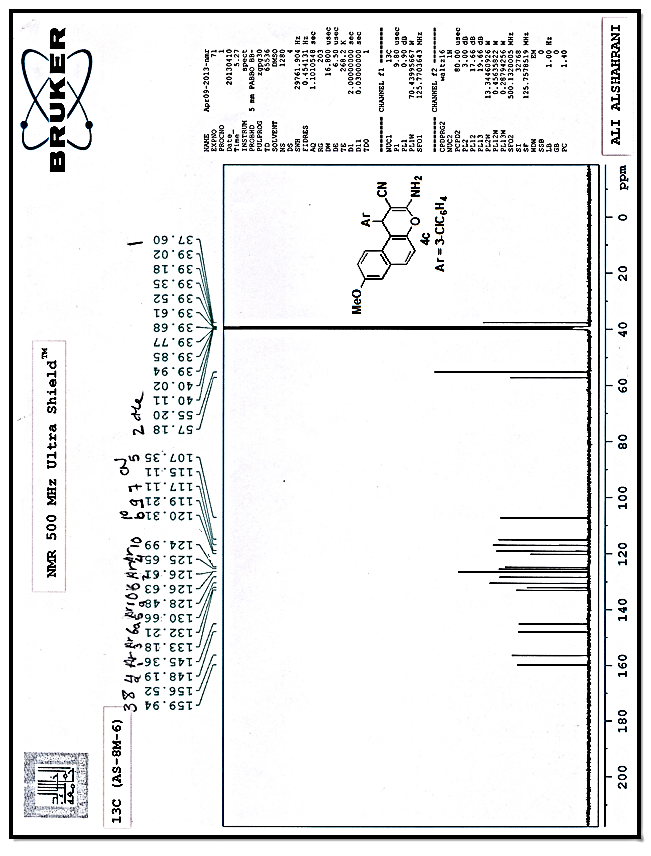


**Figure S9.** ^13^C-NMR spectrum (DMSO-*d_6_*, 125 MHz) of compound **4c.**


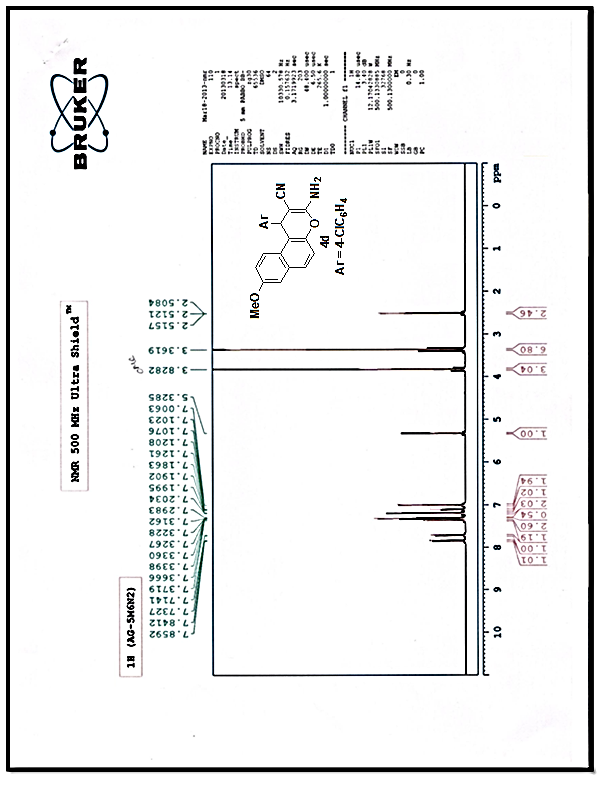


**Figure S10.** ^1^H-NMR spectrum (DMSO-*d_6_*, 500 MHz) of compound **4d.**


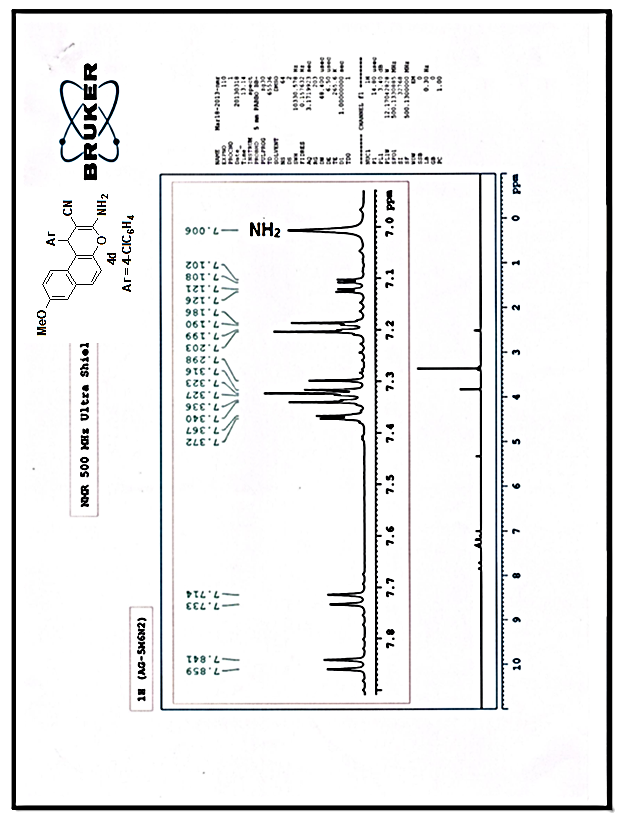


**Figure S11.** Enlarged ^1^H-NMR spectrum (DMSO-*d_6_*, 500 MHz) of compound **4d.**


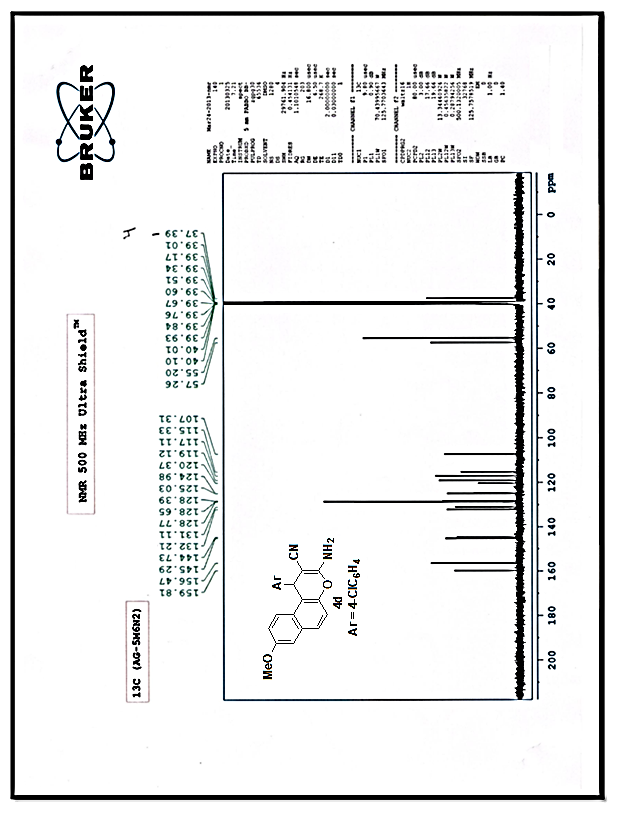


**Figure S12.** ^13^C-NMR spectrum (DMSO-*d_6_*, 125 MHz) of compound **4d.**


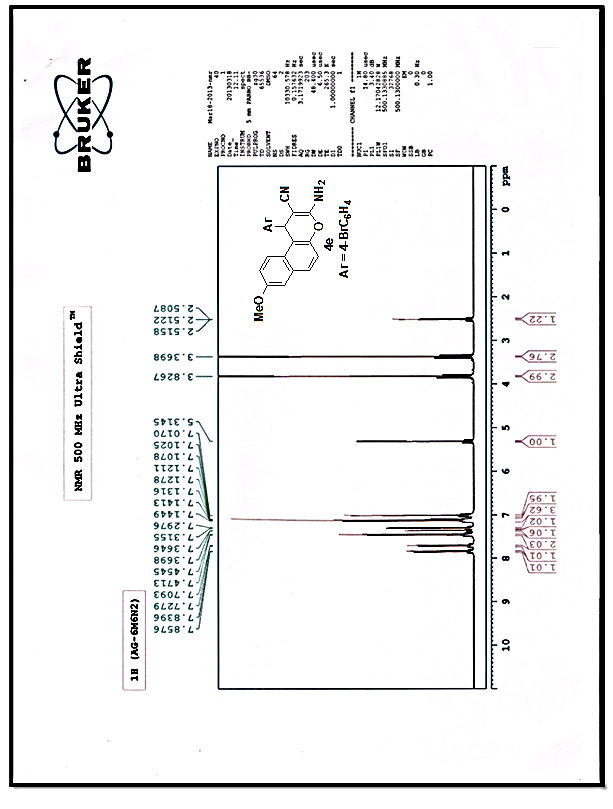


**Figure S13.** ^1^H-NMR spectrum (DMSO-*d_6_*, 500 MHz) of compound **4e.**


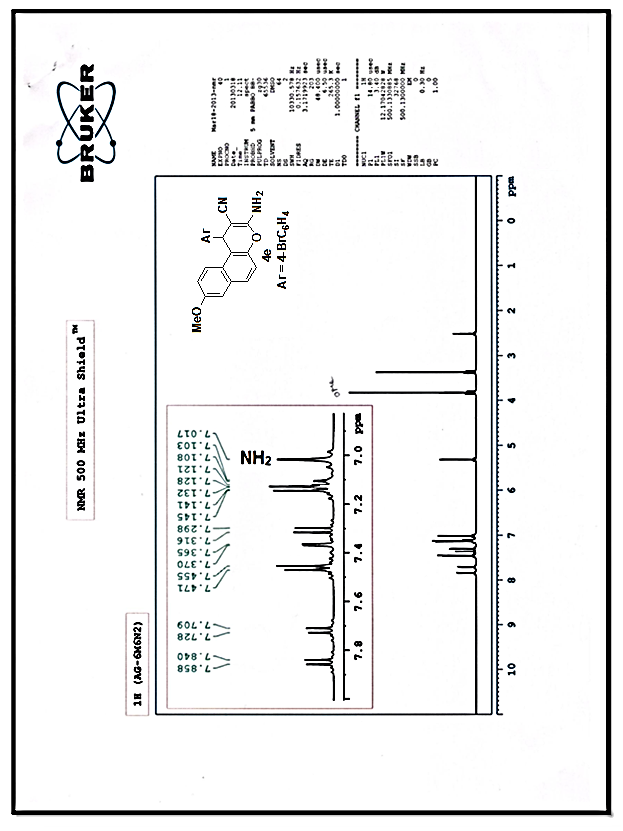


**Figure S14.** Enlarged ^1^H-NMR spectrum (DMSO-*d_6_*, 500 MHz) of compound **4e.**


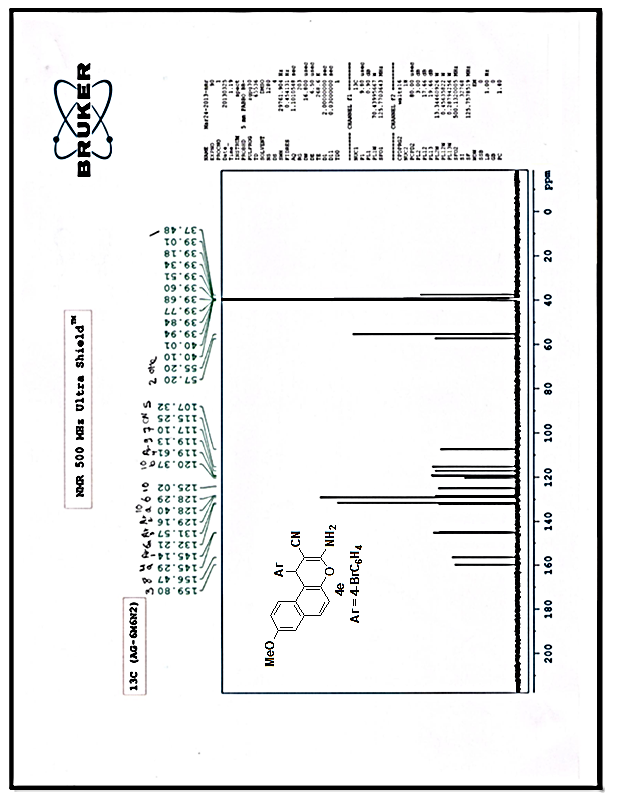


**Figure S15.** ^13^C-NMR spectrum (DMSO-*d_6_*, 125 MHz) of compound **4e.**


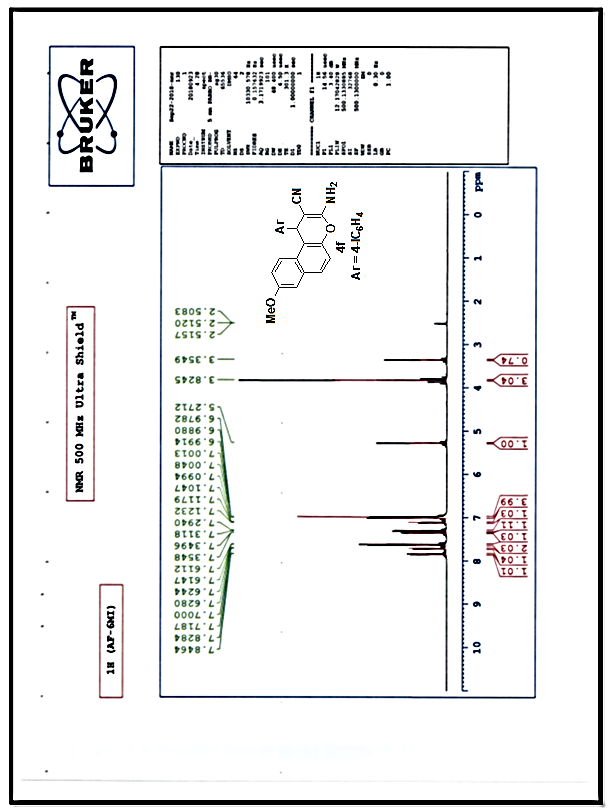


**Figure S16.** ^1^H-NMR spectrum (DMSO-*d_6_*, 500 MHz) of compound **4f.**


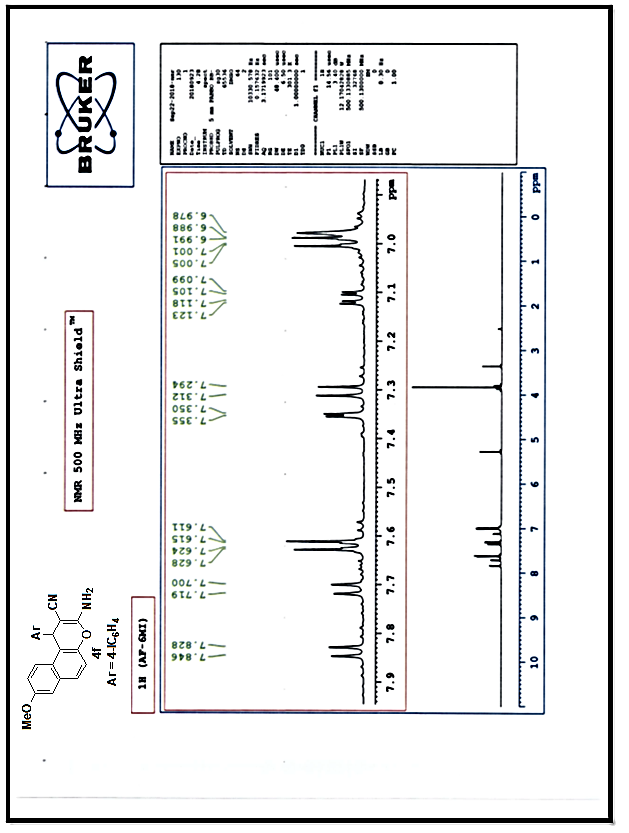


**Figure S17.** Enlarged ^1^H-NMR spectrum (DMSO-*d_6_*, 500 MHz) of compound **4f.**


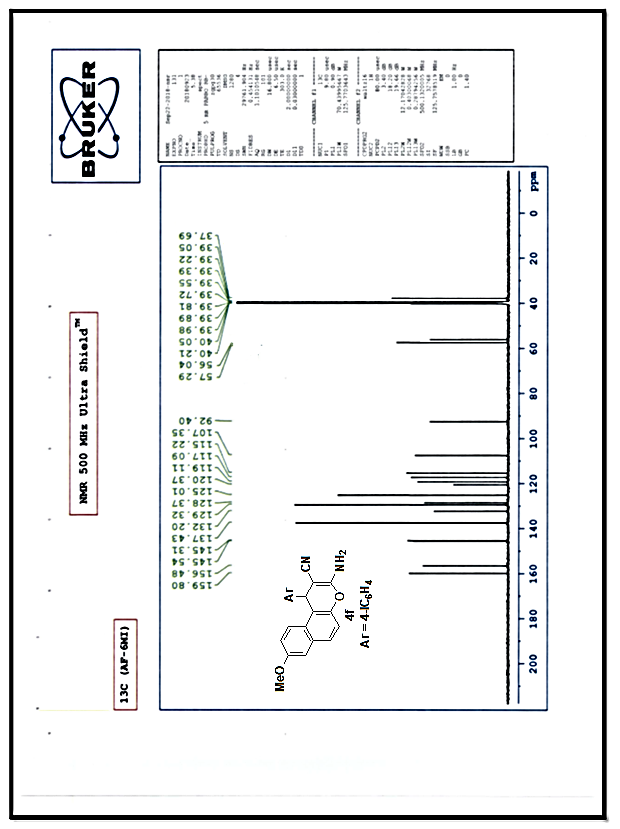


**Figure S18.** ^13^C-NMR spectrum (DMSO-*d_6_*, 125 MHz) of compound **4f.**


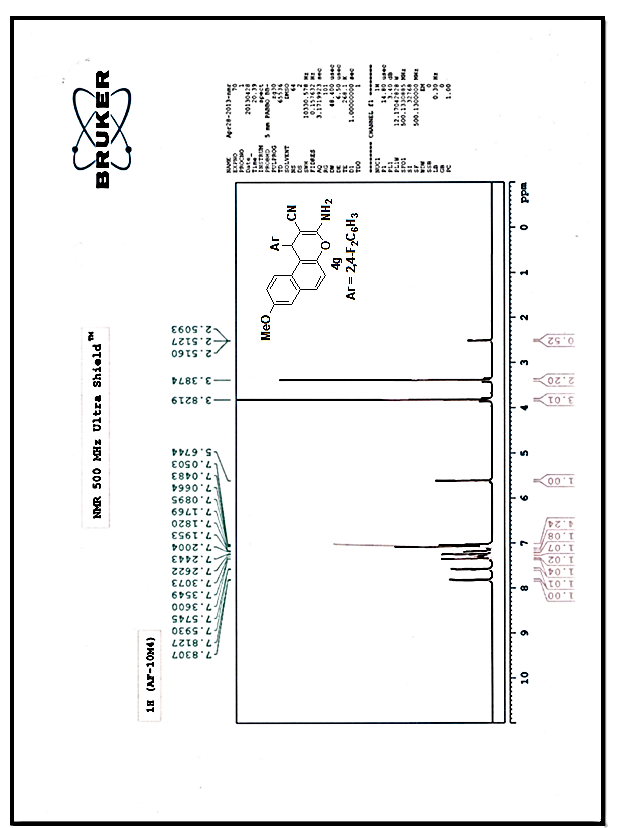


**Figure S19**. ^1^H-NMR spectrum (DMSO-*d_6_*, 500 MHz) of compound **4g.**


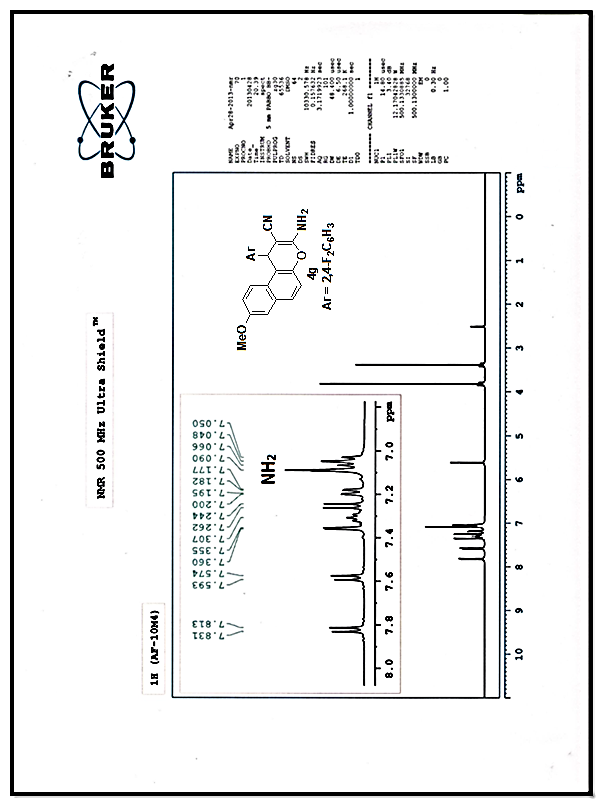


**Figure S20**. Enlarged ^1^H-NMR spectrum (DMSO-*d_6_*, 500 MHz) of compound **4g.**


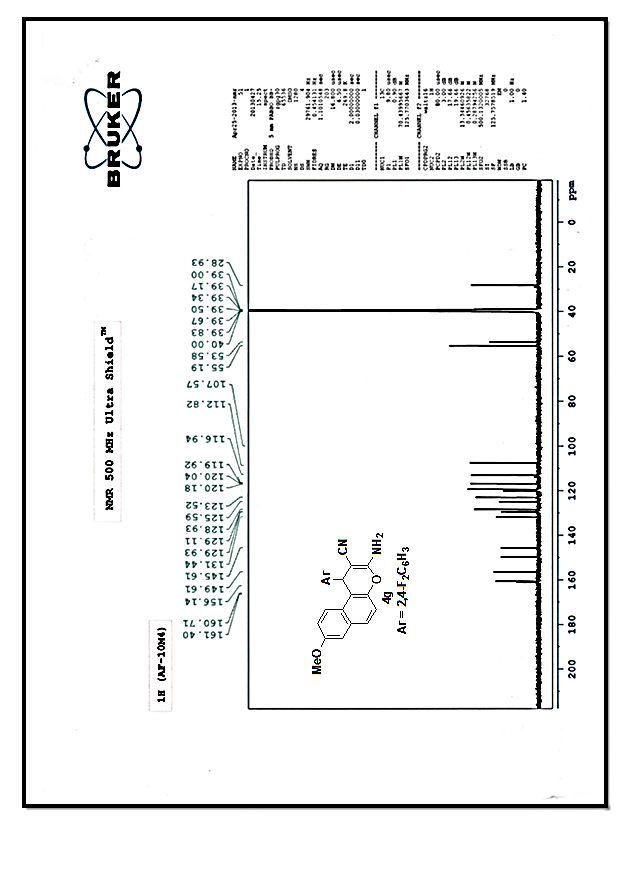


**Figure S21.** ^13^C-NMR spectrum (DMSO-*d_6_*, 125 MHz) of compound **4g.**


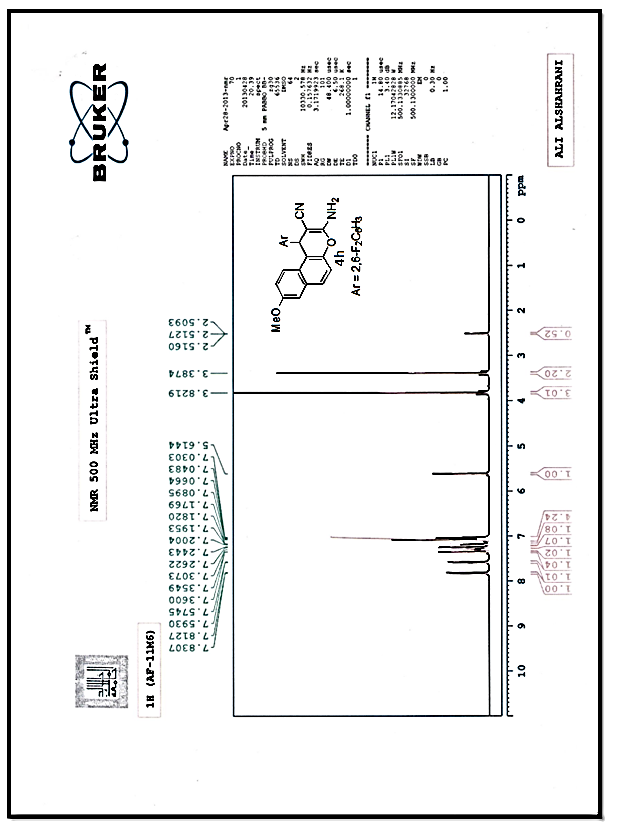


**Figure S22.** ^1^H-NMR spectrum (DMSO-*d_6_*, 500 MHz) of compound **4h.**


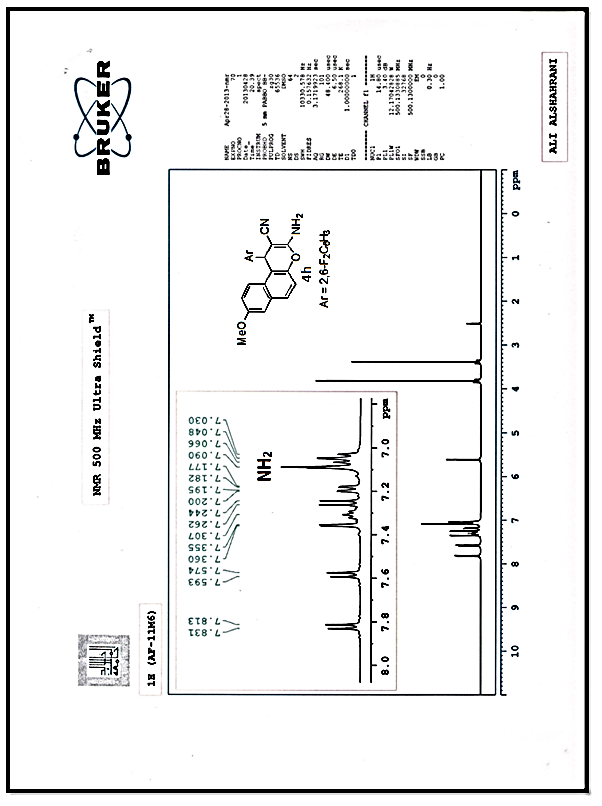


**Figure S23.** Enlarged ^1^H-NMR spectrum (DMSO-*d_6_*, 500 MHz) of compound **4h.**


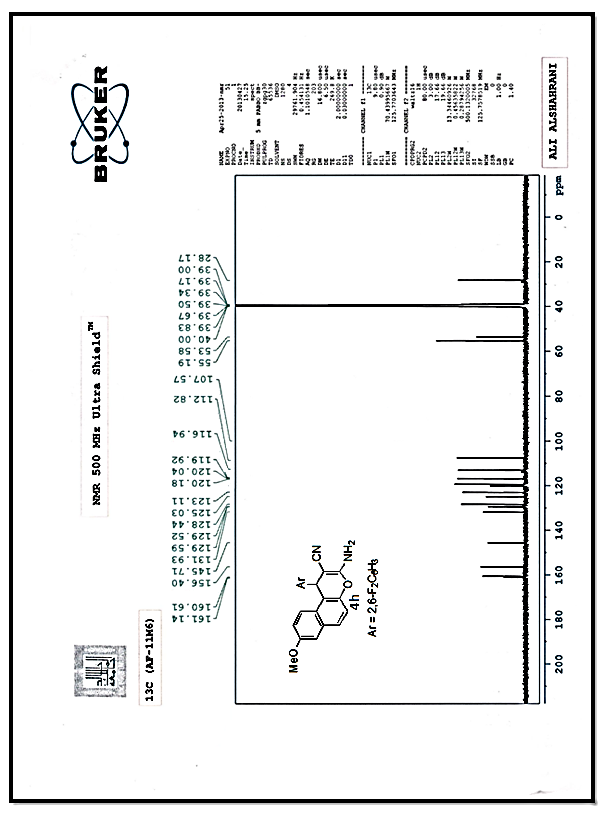


**Figure S24.** ^13^C-NMR spectrum (DMSO-*d_6_*, 125 MHz) of compound **4h.**


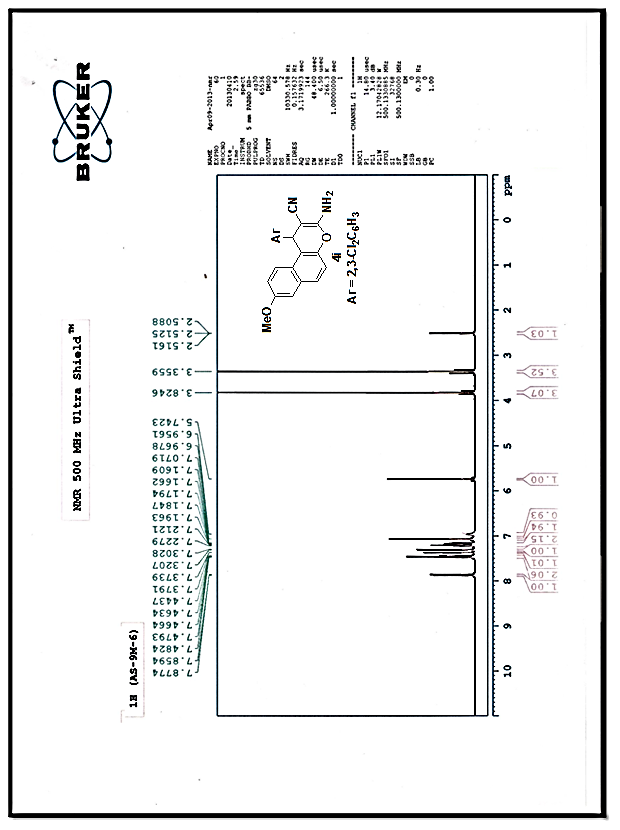


**Figure S25.** ^1^H-NMR spectrum (DMSO-*d_6_*, 500 MHz) of compound **4i.**


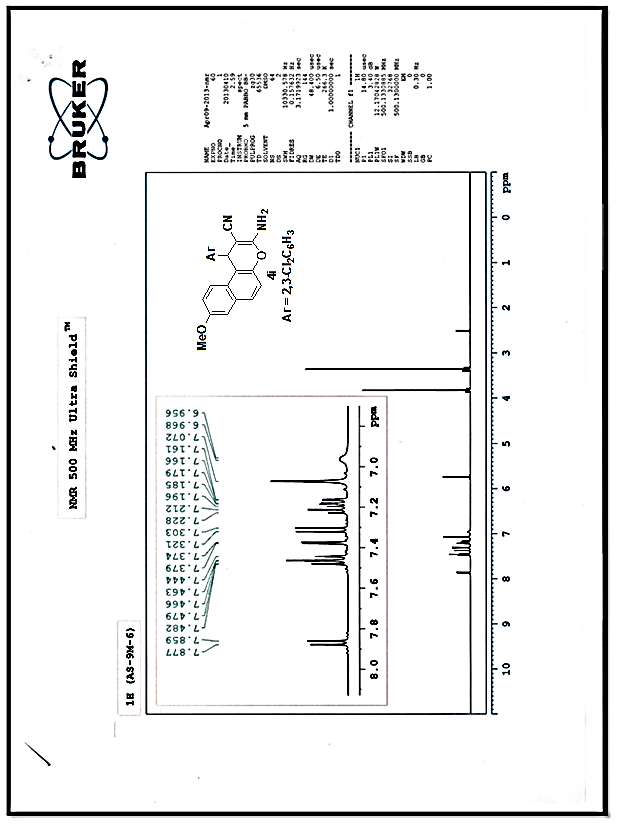


**Figure S26.** Enlarged ^1^H-NMR spectrum (DMSO-*d_6_*, 500 MHz) of compound **4i.**


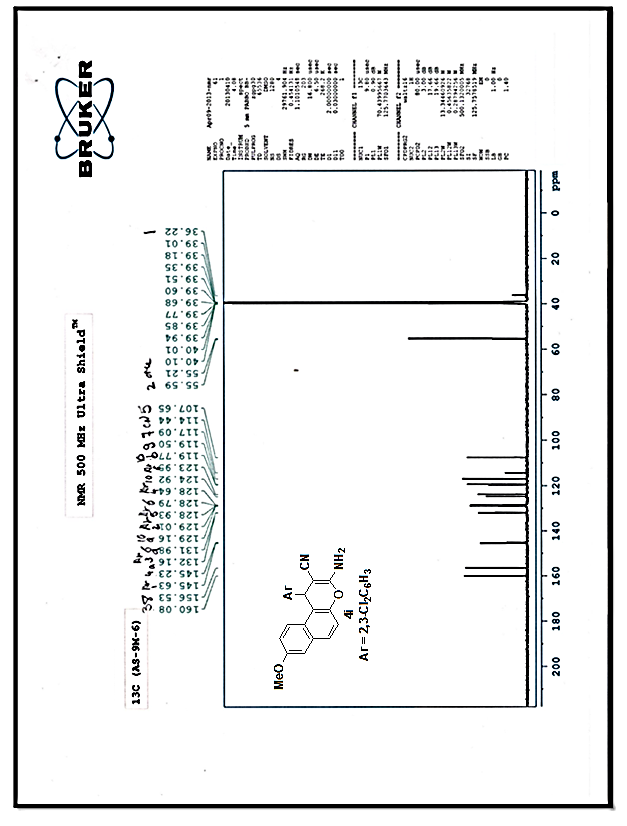


**Figure S27.** 13C-NMR spectrum (DMSO-*d_6_*, 125 MHz) of compound **4i.**


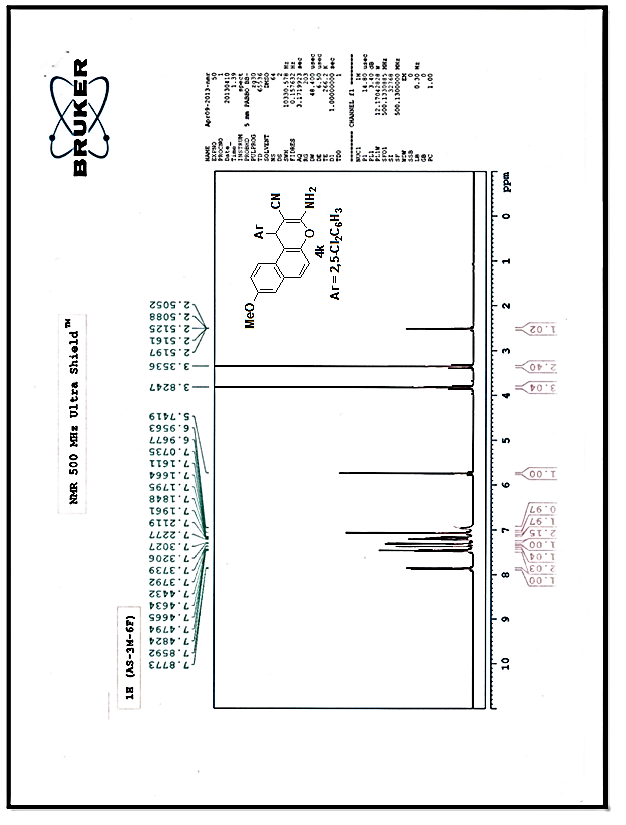


**Figure S28.** ^1^H-NMR spectrum (DMSO-*d_6_*, 500 MHz) of compound **4k.**


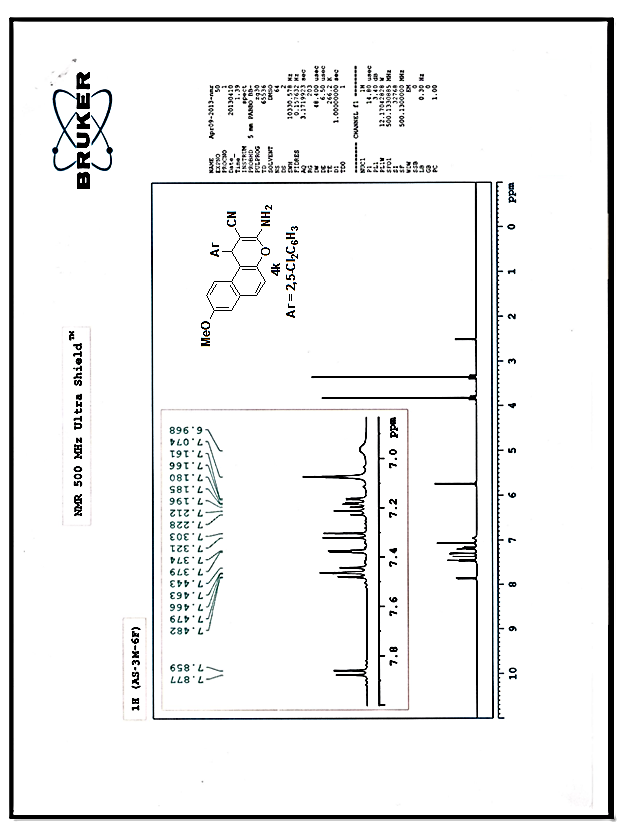


**Figure S29.** Enlarged ^1^H-NMR spectrum (DMSO-*d_6_*, 500 MHz) of compound **4k.**


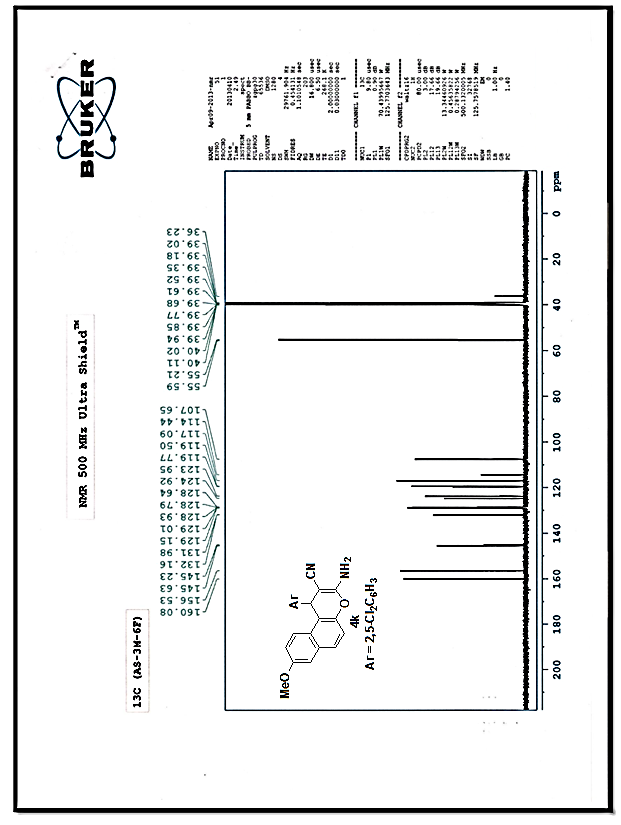


**Figure S30.** ^13^C-NMR spectrum (DMSO-*d_6_*, 125 MHz) of compound **4k.**


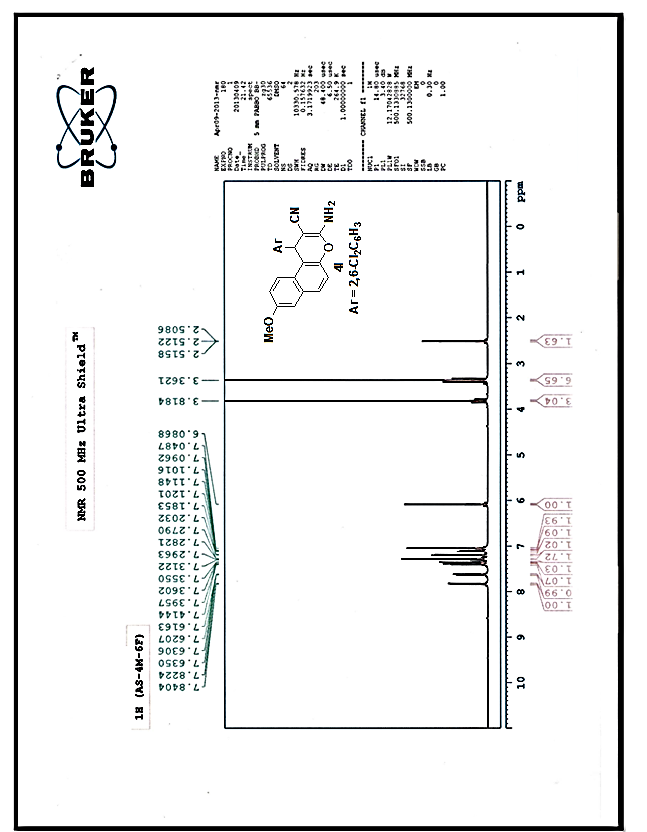


**Figure S31.** ^1^H-NMR spectrum (DMSO-*d_6_*, 500 MHz) of compound **4l.**


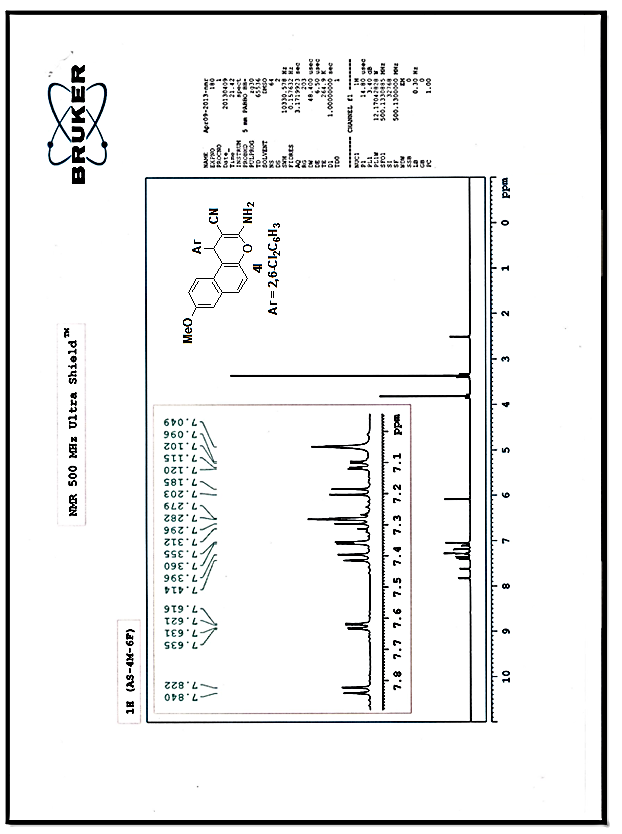


**Figure S32.** Enlarged ^1^H-NMR spectrum (DMSO-*d_6_*, 500 MHz) of compound **4l.**


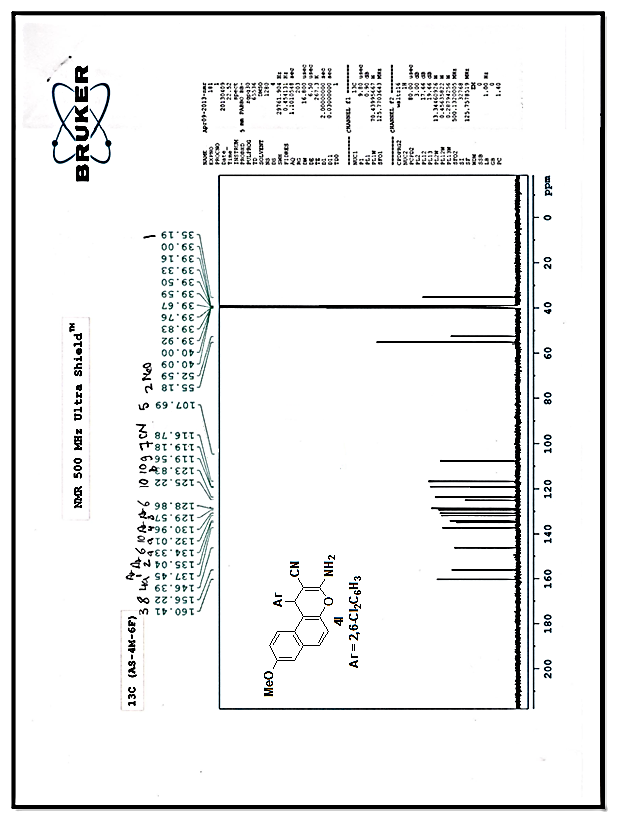


**Figure S33**. ^13^C-NMR spectrum (DMSO-*d_6_*, 125 MHz) of compound **4l.**


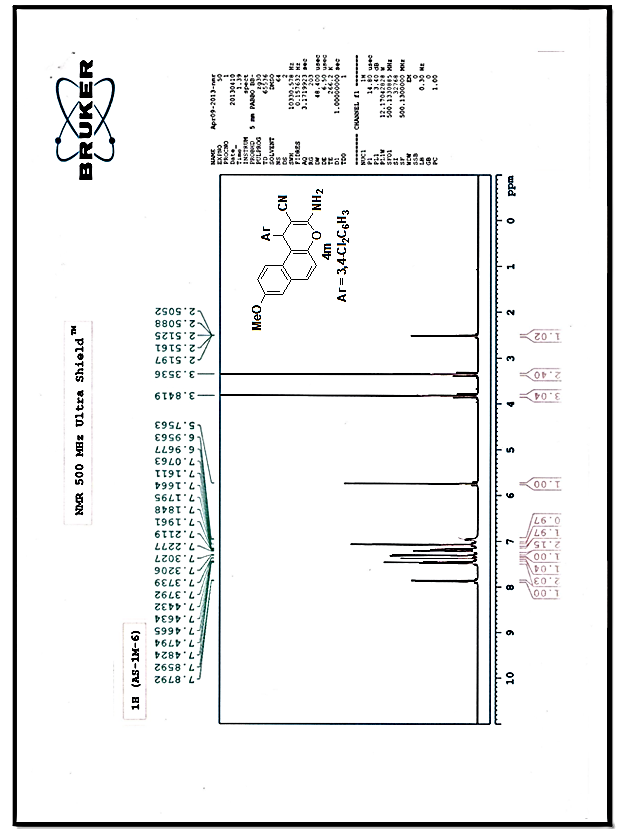


**Figure S34.** ^1^H-NMR spectrum (DMSO-*d_6_*, 500 MHz) of compound **4m.**


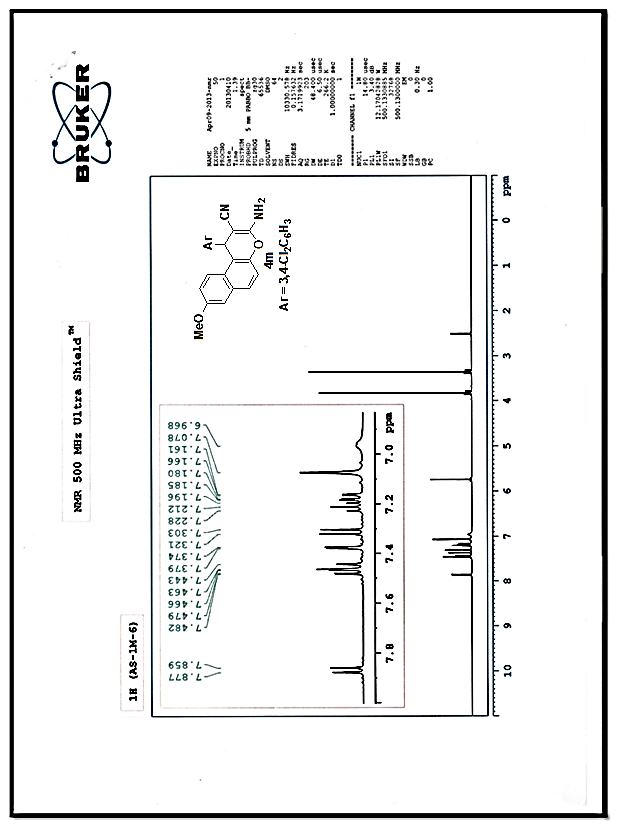


**Figure S35.** Enlarged ^1^H-NMR spectrum (DMSO-*d_6_*, 500 MHz) of compound **4m.**


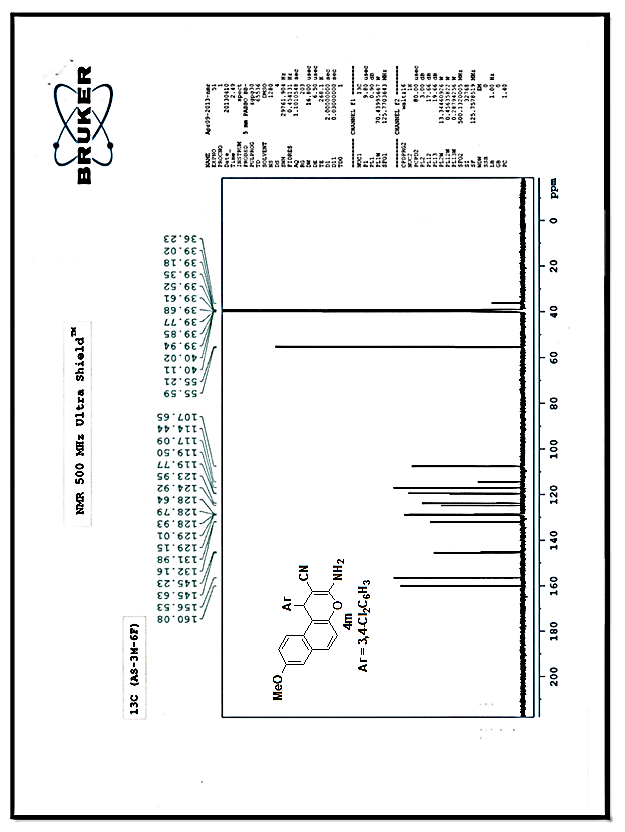


**Figure S36.** ^13^C-NMR spectrum (DMSO-*d_6_*, 125 MHz) of compound **4m.**


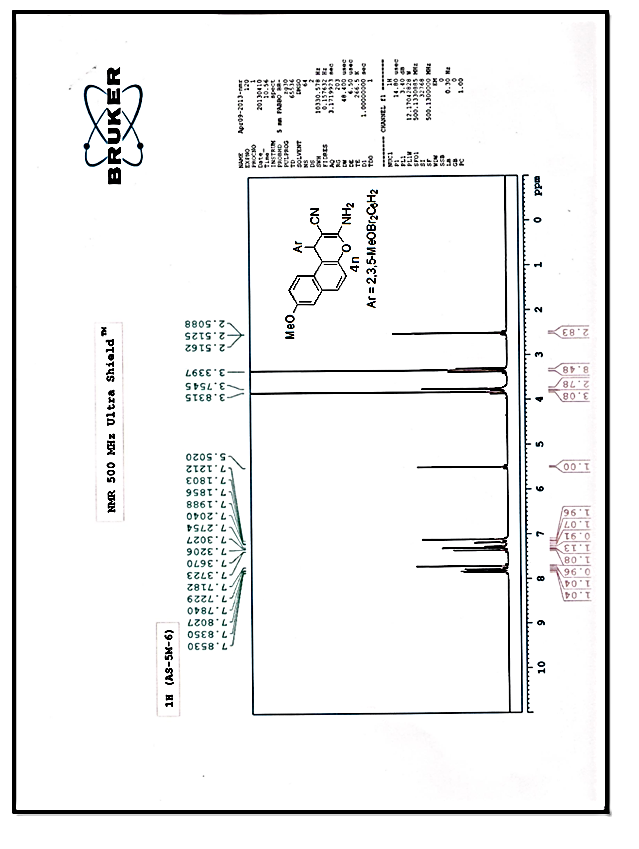


**Figure S37.** ^1^H-NMR spectrum (DMSO-*d_6_*, 500 MHz) of compound **4n.**


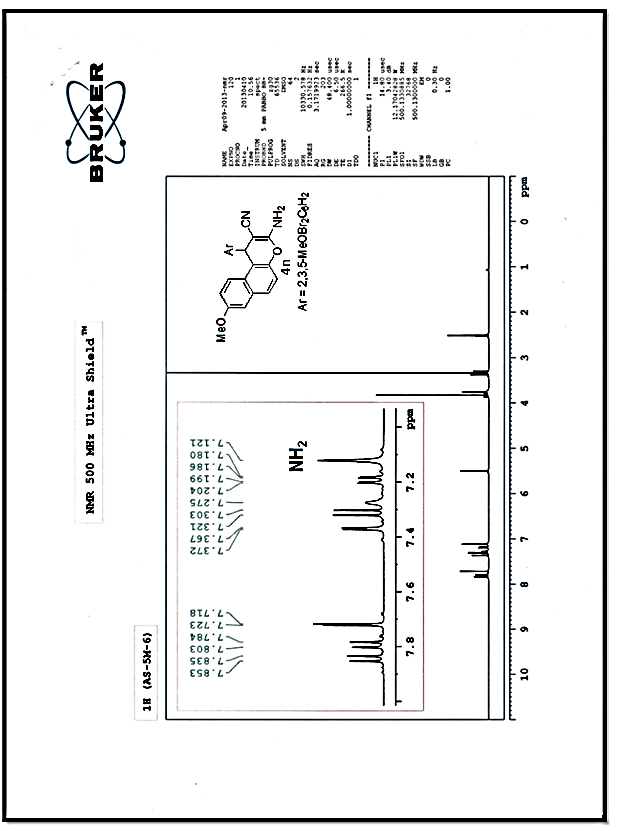


**Figure S38**. Enlarged ^1^H-NMR spectrum (DMSO-*d_6_*, 500 MHz) of compound **4n.**


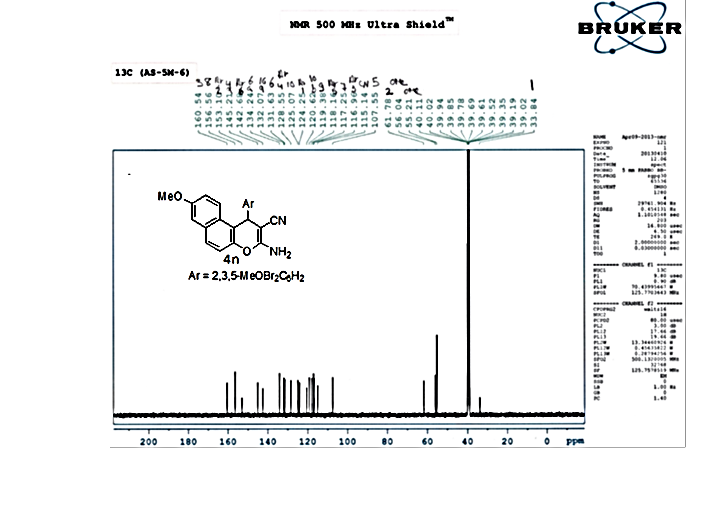


**Figure S39.** ^13^C-NMR spectrum (DMSO-*d_6_*, 125 MHz) of compound **4n.**


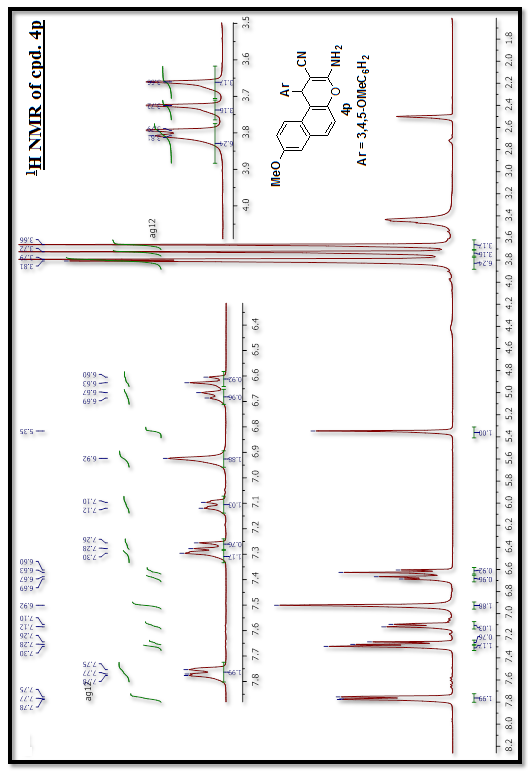


**Figure S40.** ^1^H-NMR spectrum (DMSO-*d_6_*, 500 MHz) of compound **4p.**


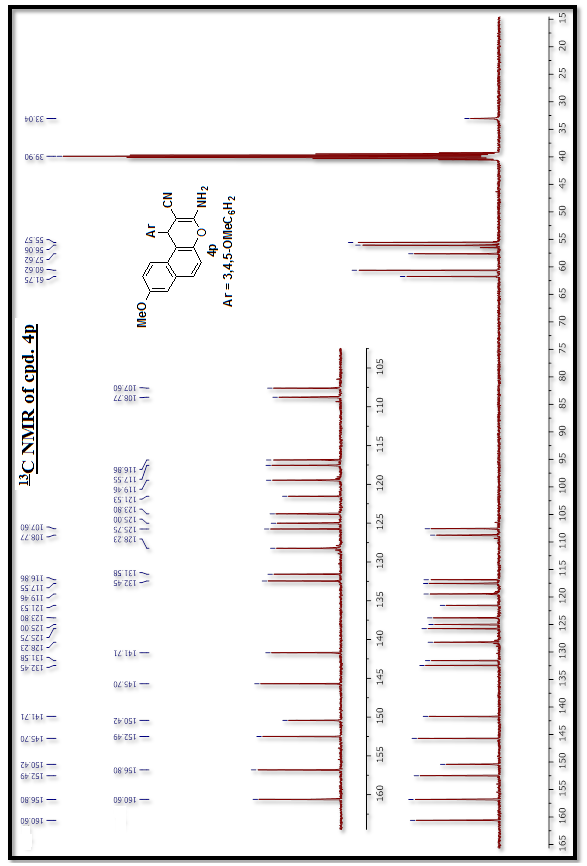


**Figure S41.** ^13^C-NMR spectrum (DMSO-*d_6_*, 125 MHz) of compound **4p.**


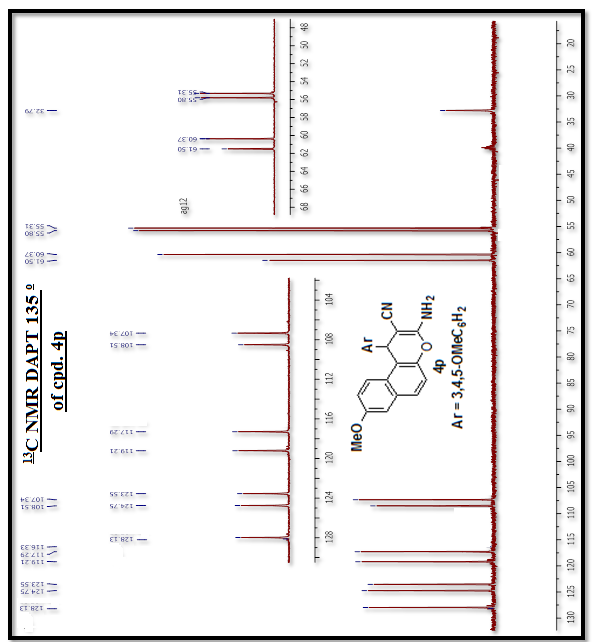


**Figure S42.** DEPT spectrum (DMSO-*d_6_*, 125 MHz) of compound **4p.**


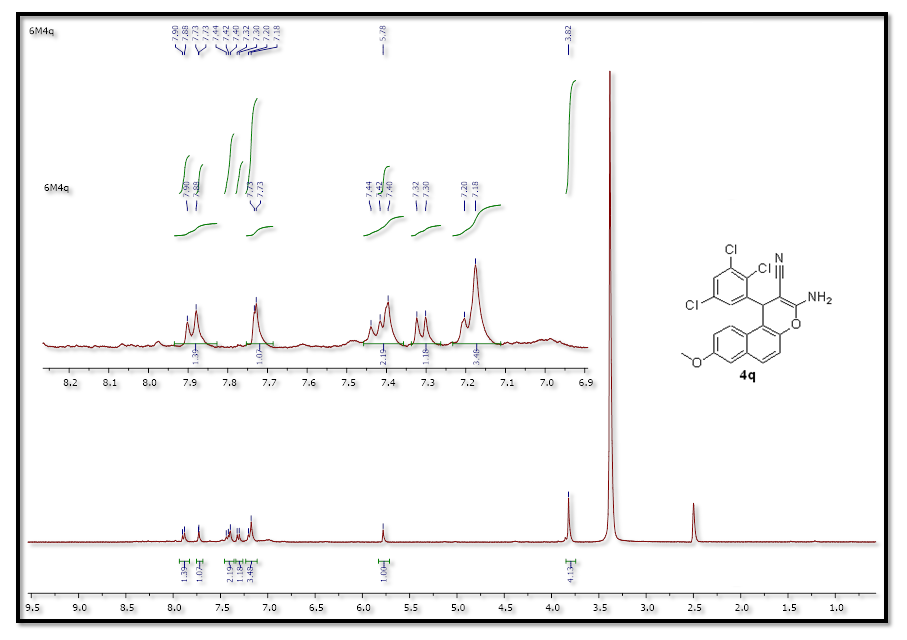


**Figure S43.** ^1^H-NMR spectrum (DMSO-*d_6_*, 500 MHz) of compound **4q.**


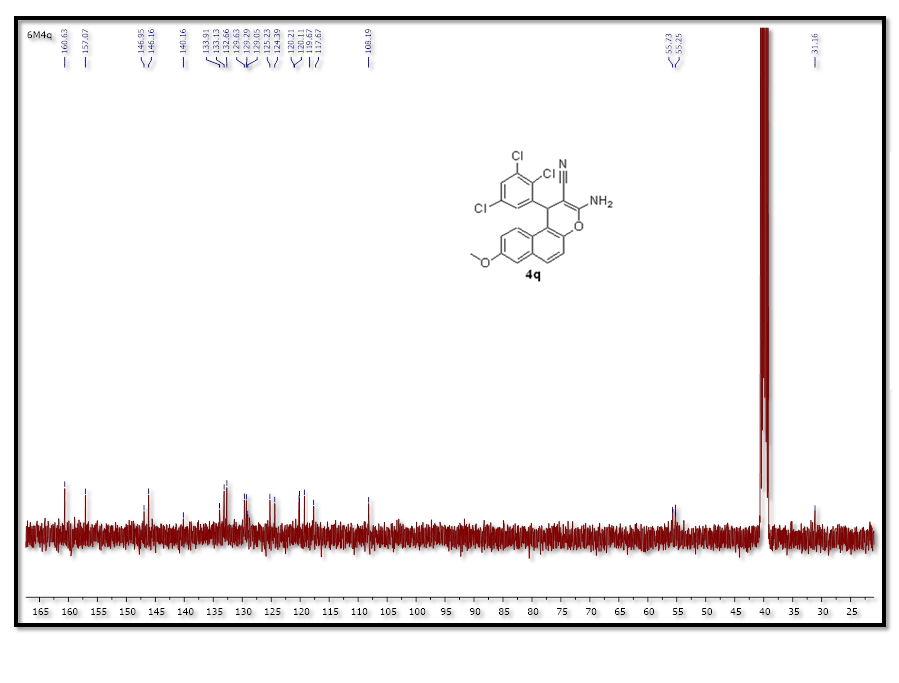


**Figure S44**. ^13^C-NMR spectrum (DMSO-*d_6_*, 125 MHz) of compound **4q.**


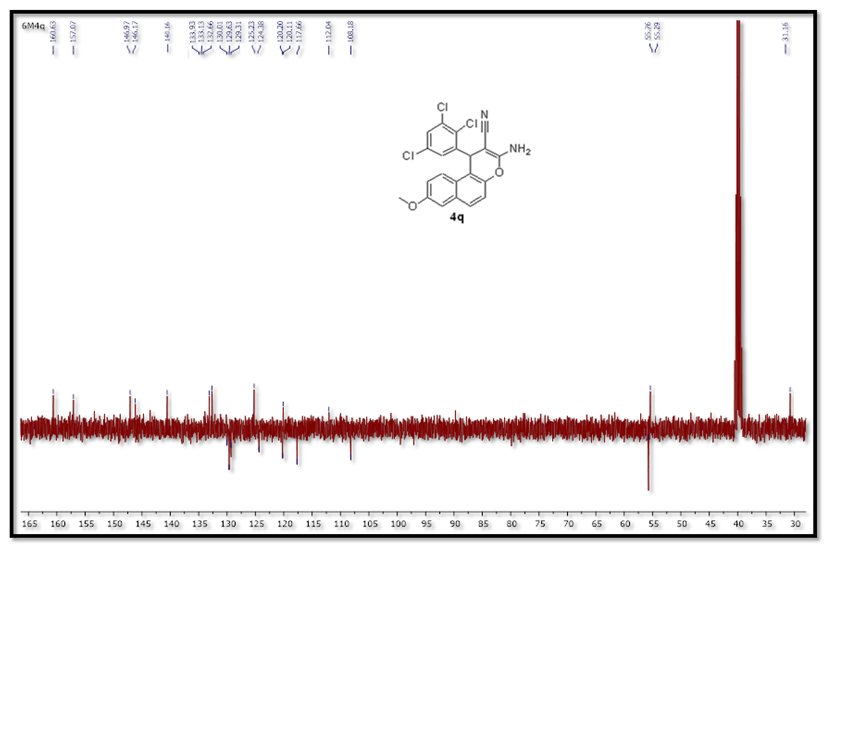


**Figure S45.** APT spectrum (DMSO-*d_6_*, 125 MHz) of compound **4q.**


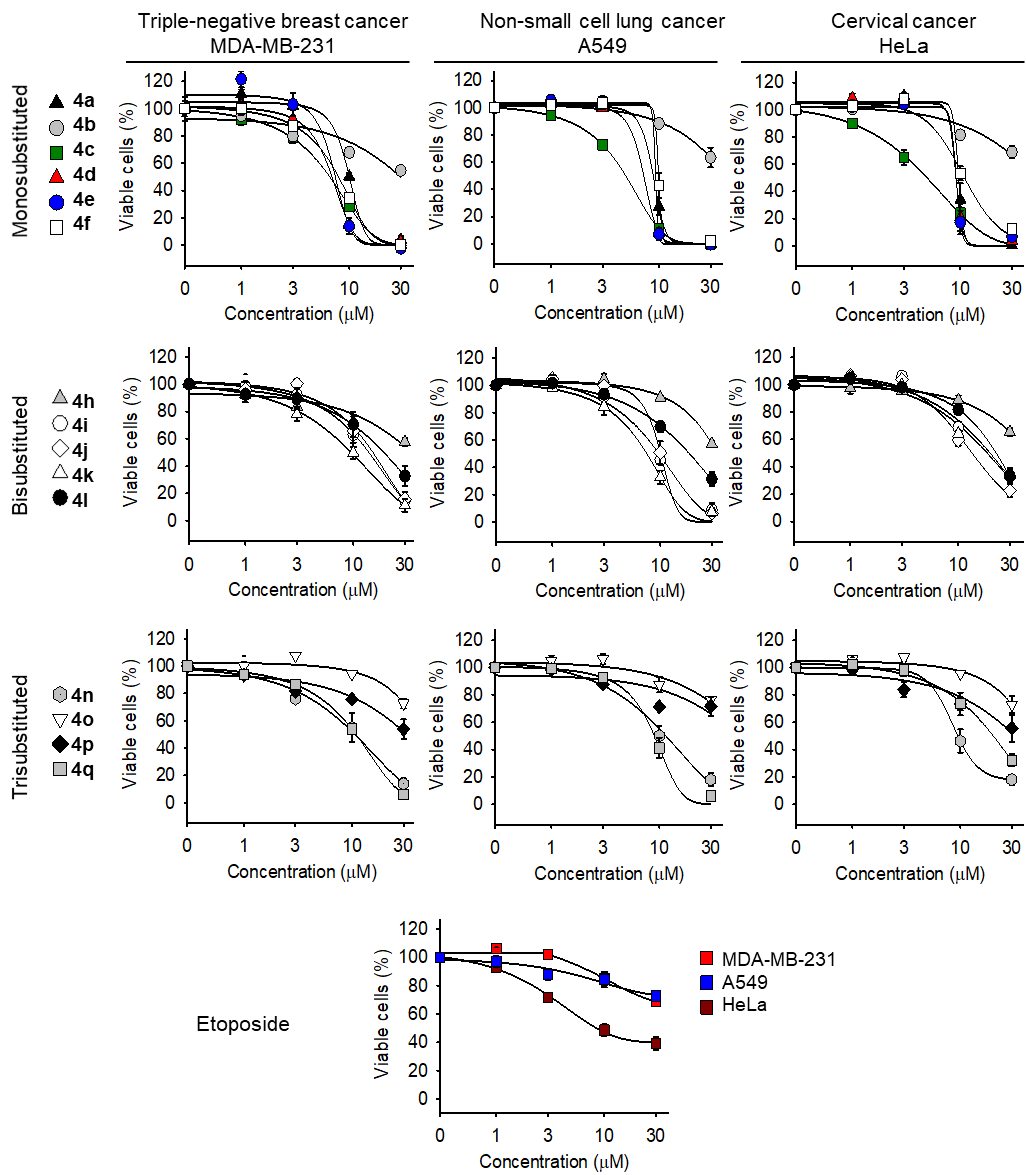


**Figure S46.** Cytotoxic activity of synthesized benzochromene derivatives against cancer cell lines MDA-MB-231, A549, and HeLa. The synthesized benzochromene derivatives with monohalogenated phenyl ring (**4a**, **4c**-**4f**) show the strongest cytotoxic activity against the tested cancer cell lines. Cancer cells were treated with various concentrations of synthesized benzochromene derivatives for 24 h. Cell viability was analyzed by XTT assay. All data are mean ± SEM, n = 3. Etoposide served as a positive control.


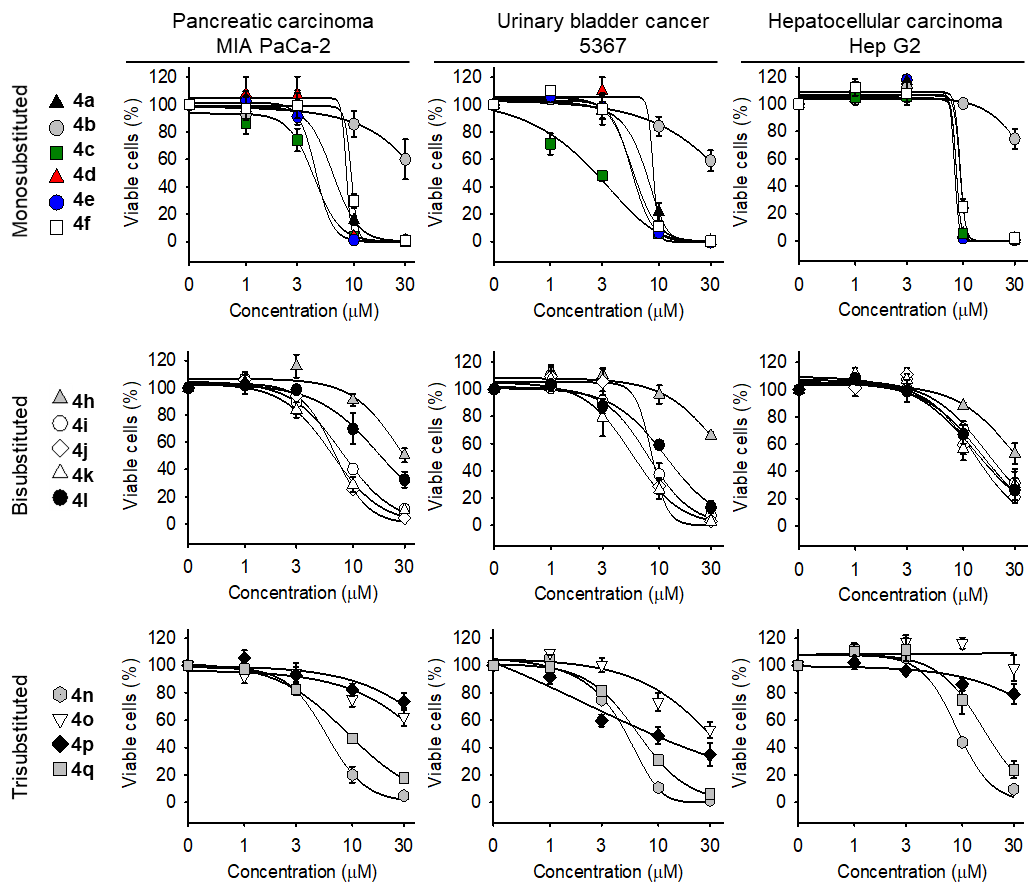


**Figure S47.** Cytotoxic activity of synthesized benzochromene derivatives against cancer cell lines MIA PaCa-2, 5367, and Hep G2. The synthesized benzochromene derivatives with monohalogenated phenyl ring (**4a**, **4c**-**4f**) show the strongest cytotoxic activity against the tested cancer cell lines. Cancer cells were treated with various concentrations of synthesized benzochromene derivatives for 24 h. Cell viability was analyzed by XTT assay. All data are mean ± SEM, n = 3.
